# Discovery of novel acridine based inhibitors of Bruton’s tyrosine kinase (BTK) via pharmacophore modeling and machine learning-driven virtual screening followed by molecular dynamic studies

**DOI:** 10.1007/s10822-026-00912-4

**Published:** 2026-09-01

**Authors:** Rand Shahin, Sawsan Jaafreh, Iman Mansi, Rufaida Al Zoubi, Bashaer Abu-Irmaileh, Yusra Azzam, Salma Azzam

**Affiliations:** 1 Department of Pharmaceutical Chemistry, Faculty of Pharmaceutical Sciences, The Hashemite University, P.O. Box 330117, Zarqa, 13133 Jordan; 2 Department of Chemistry, Faculty of Science, The Hashemite University, P.O. Box 330117, Zarqa, 13133 Jordan; 3 Department of Clinical Pharmacy and Pharmacy Practice, Faculty of Pharmaceutical Sciences, The Hashemite University, Zarqa, Jordan; 4 Department of Medicinal Chemistry and Pharmacognosy, Faculty of Pharmacy, Jordan University of Science & Technology, Irbid, Jordan; 5 Hamdi Mango Center, The University of Jordan, Amman, Jordan; 6 Jacobs School of Medicine and Biomedical Sciences, University at Buffalo, 955 Main Street, Buffalo, NY 14203 USA

**Keywords:** Bruton's tyrosine kinase, BTK, Lymphoma, Acridine, KNIME, Machine learning, Pharmacophore modeling, Virtual screening, Drug discovery

## Abstract

Bruton’s Tyrosine Kinase (BTK) has been recently recognized as an important drug design target for treating B-cell malignancies. Unfortunately, drug resistance is making the treatment of B-cell malignancies challenging. In this study, we employed a dual machine learning drug design approach that involves two parallel branches: the QSAR-GFA modeling (Branch 1) which engages the development of an interpretable structure activity relationships, and the KNIME® based machine learning modeling (Branch 2) which aimed at achieving high predictive accuracy. Subsequently, six machine learning modules, encompassing; the Random Forest model, the XGBoost Model (XGBoost), the Naive Bayes model, the k-Nearest Neighbors model, and the Support Vector Machines model were applied. Later, experimental validation through cytotoxicity assays against Raji lymphoma and K562 leukemia cell lines revealed several compounds that are exhibiting notable biological activity. For instance, compounds 166 and 168 which are acridine based displayed IC_50_ values of 0.087 and 4.92 µM in Raji cells, respectively. Also, hits 166 and 168 were subjected to enzymatic evaluation by using a radiometric HotSpot™ kinase assay against BTK and yielded an IC_50_ values of 12.2 µM and 23.8 µM, respectively. Finally, a key outcome of this study is the identification of a chemically distinct and novel, acridine-based antiproliferative hit candidates with measurable BTK inhibitory activity.

**Supplementary Information:**

The online version contains supplementary material available at https://doi.org/10.1007/s10822-026-00912-4.

## Introduction

Recently, Bruton's Tyrosine Kinase (BTK) has emerged as a drug design target for B-cell malignancies and autoimmune diseases [1]. Also, B-cell receptor (BCR) is a cell-surface receptor complex that plays a crucial role during the activation of the BTK signaling pathway [2]. Consequently, aberrant BTK signaling is implicated in the pathogenesis of many hematological malignancies, including mantle cell lymphoma (MCL), chronic lymphocytic leukemia (CLL), and Waldenström’s macroglobulinemia (WM), as well as autoimmune disorders such as systemic lupus erythematosus and rheumatoid arthritis [3]. All in all, this establishes and validates the role of BTK in B-cell related disease which has firmly recognized it as a high priority target for therapeutic intervention during the drug design process [4].

Furthermore, the clinical application and efficacy of BTK inhibitors is revealed by first generation (ibrutinib) and second generation (acalabrutinib, zanubrutinib) irreversible inhibitors, alongside with the recent integration of the first approved third generation reversible inhibitor, pirtobrutinib [2]. Therefore, the anti-BTK drugs have demonstrated remarkable clinical efficacy by treating patients with related malignancies [5]. However, despite their successes, current BTK inhibitors are still suffering from limitations regarding their selectivity, and this leads to off-target side effects such as atrial fibrillation and bleeding complications [6]. Furthermore, one of the most complex challenges that is facing the long-term use of BTK inhibitors in the market is the development of resistance mechanisms, particularly those that are related to the emergence of mutations in the amino acid Cys481in the active site of BTK which prevents covalent inhibitor from binding to the ATP active site [7]. Thus, these limitations show the importance of continuing the scientific efforts toward the development of novel BTK inhibitors that are characterized by enhanced selectivity and improved resistance profiles [8].

**Fig. 1:**
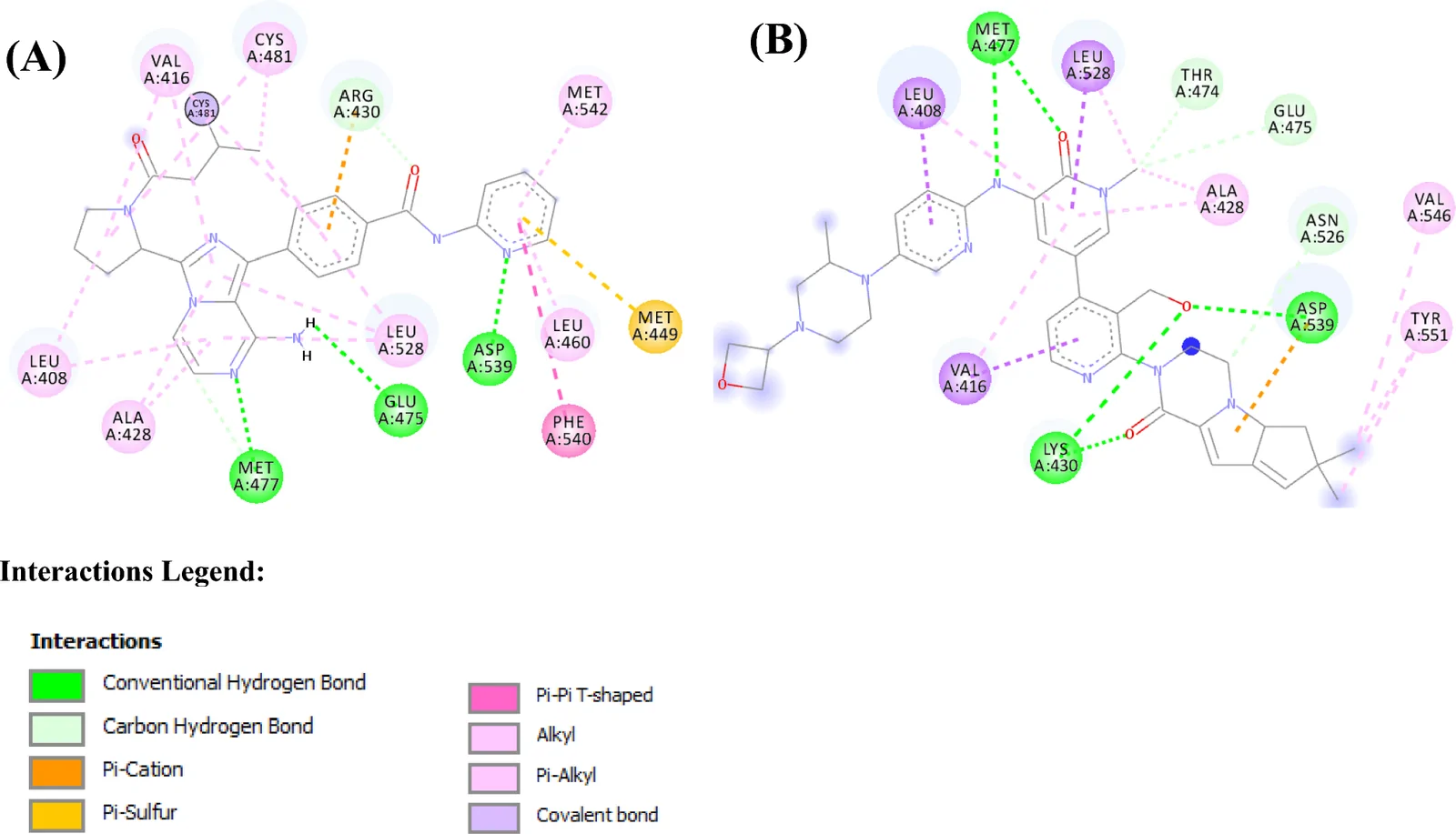
Co-crystallized poses of BTK inhibitors **A** Acalabrutinib-BTK Binding Interactions (PDB ID: 8FD9). This 2D diagram illustrates the covalent binding pose and interactions of acalabrutinib within the BTK active site. Hydrogen bonds (green dashed lines) are observed with key residues including Met 477, Lys 430, and Asp 539. Hydrophobic interactions (purple/pink dashed lines) involve multiple surrounding aliphatic and aromatic residues. A pi-anion interaction (orange dashed line) is formed with Asp 539. Fuzzy blue areas indicate favorable van der Waals contacts. Residue circles are colored based on interaction type. **B** Fenebrutinib (GDC-0853)-BTK Binding Interactions (PDB ID: 5VFI). This 2D diagram depicts the non-covalent BTK inhibitor GDC-0853's binding in the active site of the BTK kinase domain. As shown, GDC-0853 forms hydrogen bonds (green lines), multiple hydrophobic contacts (pink lines. Other interactions include those with Arg 430 and Met 449 (orange lines)

Moreover, the BTK binding pocket can exhibit different interactions with covalent and non-covalent inhibitors [9]. In particular, the BTK covalent inhibitors such as ibrutinib, acalabrutinib, and zanubrutinib, which are primarily targeting the amino acid cysteine (Cys481) through covalent bond formation (please refer to Fig. 1A which shows the predicted binding mode of acalabrutinib within the BTK active site (PDB ID: 8FD9). On the other side, non-covalent inhibitors such as fenebrutinib, nemtabrutinib, and pirtobrutinib can employ wider array of interactions, including hydrogen bonds with residues like the gatekeeper threonine 474 (Thr474), the glutamate 475 (Glu475), the glycine 480 (Gly480), and the aspartic acid 539 (Asp539), as well as hydrophobic interactions with the nonpolar residues in addition to electrostatic and π–cation interactions [4, 9, 10]. These interacting patterns are illustrated in Fig. 1B for Fenebrutinib (GDC-0853) (PDB ID: 5VFI) which emphasizes the contacts with Met477, Lys430, and Asp539.

**Fig. 2:**
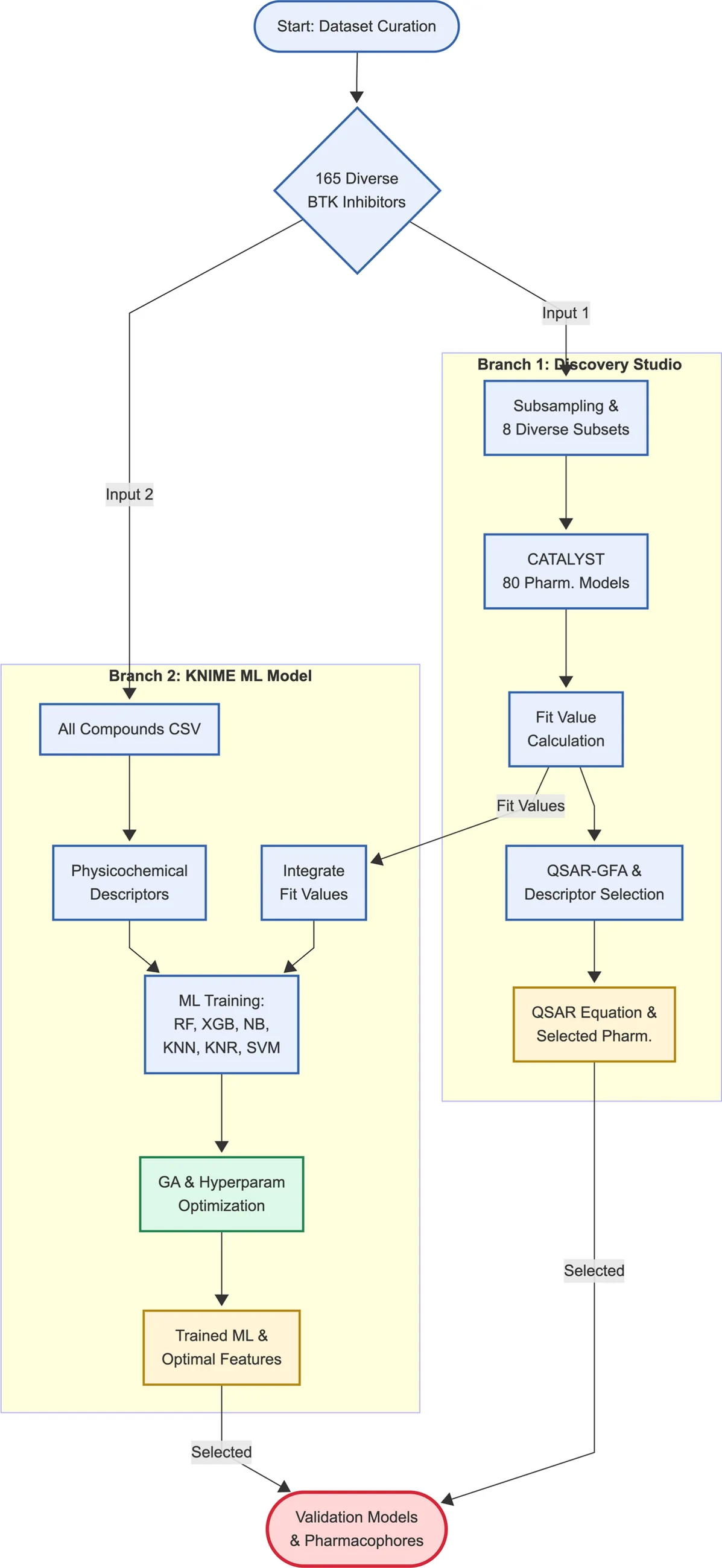
Computational Workflow for BTK Inhibitor Discovery. This flowchart illustrates the parallel two-branch computational workflow employed in this study. **Branch 1** details the Discovery Studio (DS) QSAR-GFA model development, while **Branch 2** outlines the Knime 5.4.0 Machine Learning ML-GFA model development. Both branches converge at the Validation stage, incorporating diverse validation techniques to assess model robustness and predictive accuracy, ultimately leading to the identification of novel chemotypes, including Acridine based inhibitors

Also, in this study we employed an innovative two-way computational drug design approach through applying two parallel branches: The Discovery Studio (DS) based QSAR-GFA modeling (Branch 1) which focused on developing interpretable structure activity relationships, and the KNIME based machine learning (ML) modeling (Branch 2) which aimed at achieving the highest predictive accuracy (See Fig. 2). The scientific computational drug design community has widely recognized that traditional QSAR-GFA approaches offer mechanistic interpretability by relating molecular descriptors to biological activity. However, traditional QSAR approaches are often constrained by the limited extrapolation beyond the chemical space that has been represented in the training set [11–13]. On the other side, machine learning methods are capable of capturing complex, non-linear structure activity relationships and scale them efficiently to large compound libraries, but these methods are frequently suffering from reduced interpretability, susceptibility to overfitting, and limited mechanistic transparency when used as standalone predictive tools [14]. Our dual approach aimed to provide both mechanistic insights and robust predictive models for BTK inhibitors which will ultimately lead to the identification of novel anti-BTK chemotypes. Moreover, the identified hits from this dual approach will be subsequently evaluated in vitro, and their binding modes will be analyzed by using molecular docking and SHAP based machine learning feature importance.

## Methodology

To accelerate the identification of novel inhibitors of BTK enzyme within a streamlined in-silico and in-vitro pipelines our BTK inhibitor drug design methodology was executed through the following stages:

### Dataset curation and computational workflow

This study employed an integrated computational workflow for novel BTK inhibitor discovery, beginning with collecting and structuring a carefully validated dataset of 165 diverse BTK inhibitors (See Table A in supporting material for the 2D structural representation of compounds 1–165) [15–27]. Following, in-silico preparation was employed for the compounds in Table A under supplementary material to ensure data integrity, and this included in-silico tautomer standardization and ionization state optimization by using the “Prepare Ligand” protocol in Discovery Studio version 4.5 (DS 4.5). Later, conformational analysis for the structural space of each compound was employed by using the “Generate Conformations” protocol embedded in DS 4.5 to enhance full range 3D structural representation. Then, the compiled dataset of 165 BTK inhibitors, along with their calculated molecular descriptors and pharmacophoric features, was divided into training and test subsets by using the “Generate Training and Test Data” protocol implemented in Discovery Studio 4.5 [28]. The training set was constructed to develop a QSAR model that can describe the structural and physicochemical determinants of the BTK inhibitory activity, while the test set served to independently validate the predictive quality of the resulting model. The splitting was performed by using the “Diverse Molecule Method,” which employs a predefined set of molecular properties for clustering to ensure that both subsets can represent the chemical diversity of the parent dataset. This protocol was configured to partition the 165 BTK inhibitors into an 80% training set (127 compounds) and a 20% test set (38 compounds). Subsequently, the drug discovery computational workflow was performed by using two parallel branches:

Branch 1: QSAR-GFA model development by using the DS 4.5. This branch focused on generating a QSAR model through employing the “Genetic Function Approximation” and the “Multiple Linear Regression (GFA-MLR)” protocols in DS 4.5. The set up of the GFA-MLR model involved training set assembly, testing set organization, pharmacophoric space exploration, fit value calculation, and GFA guided descriptor selection (See Fig. 2).

Branch 2: The KNIME® Machine Learning (ML) model development branch: Machine learning algorithms were employed to find an advanced predictive models by using the KNIME® analytics platform. Moreover, it is worth mentioning that the major steps that were employed during the ML stage are: the CSV data compilation step, the incorporation of physicochemical descriptors and pharmacophore fit values step, the scanning of machine learning algorithms step, and finally the feature importance analysis step (SHapley Additive exPlanations (SHAP)).

**Fig. 3:**
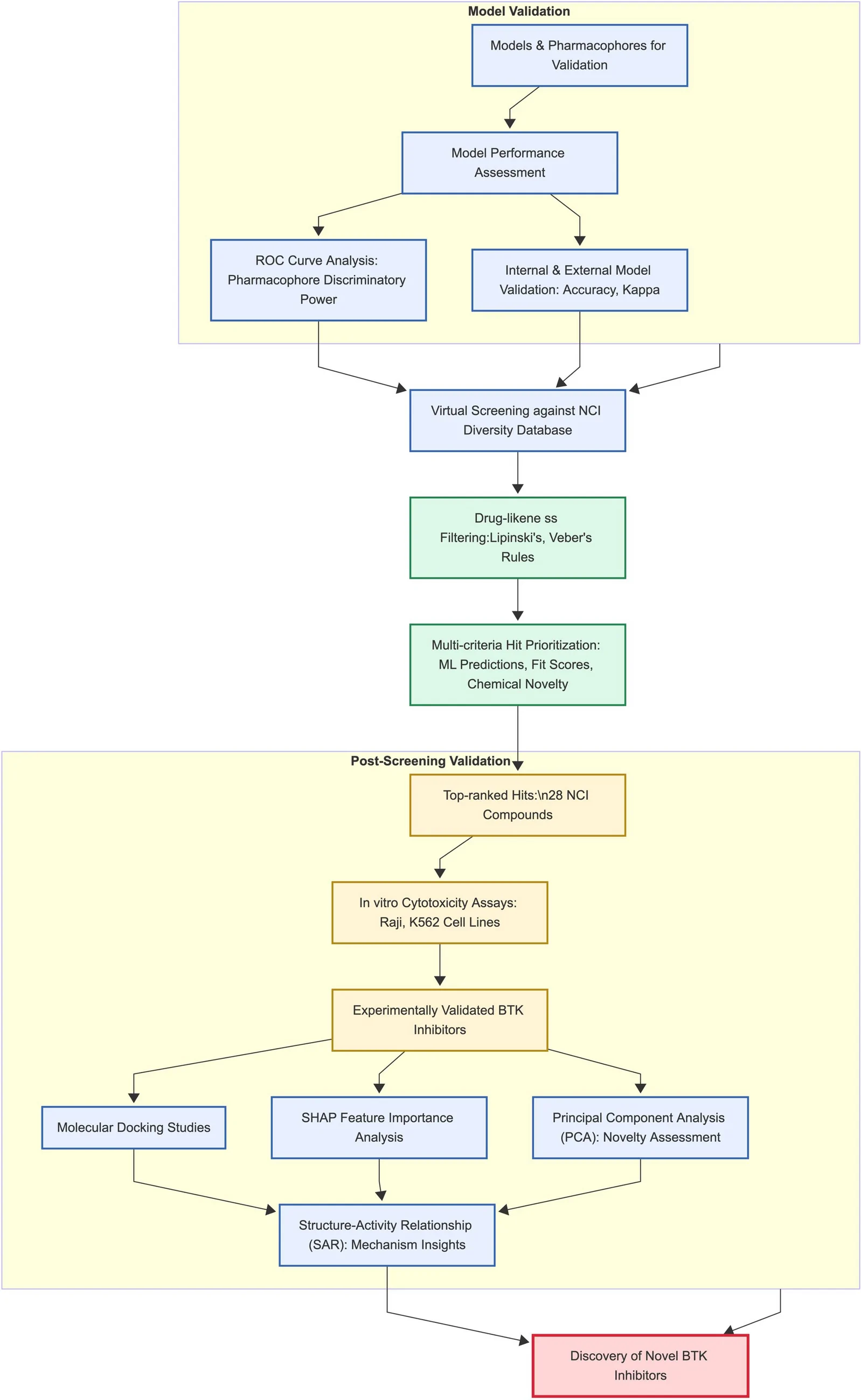
Validation flowchart for the discovery of novel BTK inhibitors. This diagram details the critical validation procedures used to assess predictive models and select top-ranked hits for experimental investigation

Finally, both branches converged for validation (see Fig. 3) by incorporating ROC curve analysis, virtual screening against the NCI set, and in-vitro experimental validation of the selected hits. The following subsections provide a detailed description of each stage of our in-silico and in-vitro pipelines.

### BTK inhibitor pharmacophoric space characterization

To elucidate the key three dimensional chemical features that govern the BTK inhibitory activity inside its binding pocket, a pharmacophore generation pipeline was implemented by using the Catalyst algorithm within the BIOVIA Discovery Studio (DS) 4.5. Thus, multiple structurally diverse training subsets spanning over at least three orders of magnitude in biological potency were employed (see supplementary for Table B). Hence, these subsets were systematically constructed to ensure adequate representation of BTK inhibitors (please refer to supplementary sections S1, S2, and S3) [28–33]. Subsequently, the resulting pharmacophoric models were validated and integrated within the QSAR (Branch 1) and the machine learning (Branch 2) workflows (See Fig. 2). Afterwards, the pharmacophoric fit values along with their calculated physicochemical descriptors, were incorporated as descriptors for activity prediction and virtual screening. A detailed information about the training set construction, pharmacophore generation and validation procedures, descriptor calculations, and fit value equations are provided in the Supplementary Information (Sections S1-S3).

### Branch 1: GFA-MLR QSAR model development for BTK inhibitors

To model a statistically predictive equation that is relating the structural and pharmacophoric descriptors of BTK inhibitors (compounds 1–165 in table A under supporting material) to their IC_50_ values, a Genetic Function Approximation combined with Multiple Linear Regression (GFA-MLR) approach was employed by using the Discovery Studio 4.5 (DS 4.5) software. The GFA-MLR protocol can establish an optimal mathematical relationship between two sets of independent variables (the pharmacophore fit values and the physicochemical molecular descriptors) with the dependent variable (IC_50_ values).

During our GFA-MLR analysis, we explored various equation forms, including linear, binary, cubic, and quadratic, and the equation length was permitted to range from 5 to 15 descriptors to ensure comprehensive model exploration. Then, to evaluate the statistical quality and the predictive power of each generated QSAR equation, a scoring system was implemented by incorporating evaluation criteria on both the training set which is used for model building and the test set which is used for external validation. The model validation adhered to the previously established OECD principles to assure the QSAR model reliability [34], alongside internal validation metrics such as Friedman Lack-of-Fit, r^2^, adjusted r^2^, and the F statistic, and external validation metrics such as predictive r^2^ (r^2^pred) and Q^2^ were employed [35].

### Branch 2: KNIME 5.4.0 machine learning model development

On the other hand, six machine learning models including, Random Forest (RF), XGBoost (Linear Model) [36], probabilistic methods (Naive Bayes), distance-based methods (k-Nearest Neighbors), neural networks (RProp Multilayer Perceptron), and Support Vector Machines (SVM) were developed and evaluated to predict the bioactivity of compounds 1–165 in table A under Supplementary Information (see Fig. 2, Branch 2)). This diverse range of algorithms encompassed ensemble, probabilistic, distance-based, and neural network approaches, allows for a comprehensive assessment of machine learning predictive performance.

#### Data preprocessing, feature selection, and hyperparameter optimization

**Fig. 4:**
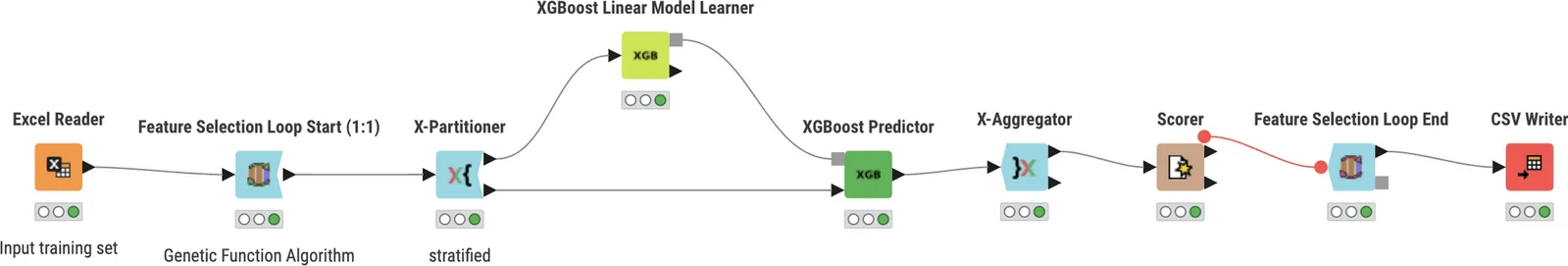
KNIME workflow segment demonstrating feature selection and model training using XGBoost Linear Model. The workflow highlights the integration of a feature selection loop Start (1:1) employing a Genetic Function Algorithm, feeding into the XGBoost model for prediction and evaluation

Our initial dataset, comprising 165 structurally diverse BTK inhibitors with their reported IC_50_ values (Table A under supplementary material), was preprocessed within KNIME (Version 5.4.0) after partitioning it to trial test (127 compounds) and test (38 compounds) (see Fig. 2, Branch 2). Thus, we employed a stratified cross-validation to explore various feature selection strategies and to evaluate the resulted model performance. Hence, our feature selection node workflow incorporated a Genetic Algorithm (GA) strategy that is already implemented in KNIME (Version 5.4.0) by using the feature selection node (see Figs. 3 and 4). And so, the GA node was configured to explore feature subsets by utilizing a lower bound of 4 and an upper bound of 8 features per subset. Also, a population size of 500 and a maximum of 100 generations were employed to ensure sufficient search within the feature space. Moreover, the iterative GA-based feature selection process was repeated five times to mitigate bias and ensures that each model was trained and tested on both the training and the test sets.

Later, we employed the tenfold stratified model evaluation strategy to ensure robust evaluation and mitigate model overfitting (Fig. 4). Hence, for each cross-validation iteration, the input dataset was partitioned row-wise into ten subsets by utilizing the X- partitioner node, then the dataset was divided into a training set and a test set. Afterward, this partitioning procedure was repeated ten times, with each subset serving as the test set in one iteration. Finally, a subset of consistently high predictive power was ultimately selected for final evaluation and the classification performance of the resulted ML models was evaluated through focusing on the model’s ability to correctly categorize compounds from the training and testing sets into “Actives” and “Non-actives”. Two metrics were employed to evaluate the success of the ML models: the accuracy metric (defined by Eq. 1 [37]) and Cohen’s Kappa metric (defined by Eq. 2 [38]).

1 $$ {\mathbf{Accuracy}} = \left( {{\mathbf{TrP}} + {\mathbf{TrN}}} \right)/{\mathbf{N}} $$

where TrP represents True Positives, or the count of "Active" compounds correctly classified as such. Then TrN represents True Negatives, or the count of "non-active" compounds correctly classified as such. And finally, N is the total number of compounds in the evaluation set. Meanwhile, Cohen’s Kappa (κ) (Eq. 2) was also used as a measure of agreement beyond chance. It is formulated as:

2 $$ {\mathbf{Cohen}}^{\prime}{\mathbf{s}} \, {\mathbf{Kappa}}\left( {{\boldsymbol{\upkappa}}} \right) = \left( {{\mathbf{Ao}} - {\mathbf{Ae}}} \right)/({\mathbf{1}} - {\mathbf{Ae}}) $$

where Ao is the Observed Agreement, is numerically equal to the accuracy and Ae stands for the Expected Chance Agreement, is the probability of agreement occurring randomly, calculated based on the marginal probabilities of each category. Usually, Cohen’s Kappa value ranges from -1 to 1. A value of 1 signifies perfect agreement but when Cohen’s Kappa acquires the value of 0 this indicates an agreement equivalent to chance, and when Cohen’s Kappa acquire negative values, this suggests agreement worse than chance prediction [38].

#### Machine learning hyperparameter optimization

The hyperparameters for each ML model were configured within KNIME (Version 5.4.0), as detailed below for each of the six classifiers. As for the RF model (KNIME Random Forest Learner), the Information Gain Ratio was selected as the splitting criterion, with 100 trees (models) employed, no maximum tree depth restriction, a minimum node size of 1, attribute sampling per split based on the square root of the total number of descriptors, and bootstrap resampling equal to the training set size for each tree; prediction was performed by using hard voting, and a static random seed was used for reproducibility. Also, for the XGBoost model (KNIME XGBoost Linear Model Learner), a linear booster with a softprob objective was used, configured with 100 boosting rounds and a base score of 0.5. Then, for the SVM model (KNIME SVM Learner), a radial basis function (RBF) kernel was selected with sigma = 0.1 and an overlapping penalty (C) = 1.0. For the k-Nearest Neighbors model (KNIME K Nearest Neighbor node), k was set to 3, Euclidean distance was used, and neighbors were weighted by distance. Furthermore, the Naive Bayes model (KNIME Naive Bayes Learner), the default probability and minimum standard deviation were each set to 0.0001, the threshold standard deviation was set to 0.0, and a maximum of 20 unique nominal values per attribute was allowed. Finally, the neural network model (KNIME RProp MLP Learner), a multilayer perceptron with one hidden layer of 10 hidden neurons was trained using the Resilient Backpropagation (RProp) algorithm for a maximum of 100 iterations, with weight initialization seeded for reproducibility; as RProp adapts per-weight step sizes internally, a single global, and the activation and loss functions are fixed internally by the KNIME implementation. Notably, for the RF, SVM, k-Nearest Neighbors, Naive Bayes, and neural network models, the same tenfold stratified cross-validation scheme (stratified on the activity class column, described in Section “Data Preprocessing, Feature Selection, and Hyperparameter Optimization”) was used for model evaluation.

#### ROC analysis for evaluating the pharmacophore model discriminatory power

To validate the selected pharmacophoric models (GFA-HypoA-T8-10, GFA-HypoE-T8-10, GFA-HypoG-T3-2, ML-HypoB-T2-5, and ML-HypoH-T8-4; Tables 1 and 3), a comprehensive multi-metric pharmacophore validation framework was implemented by using the BIOVIA Discovery Studio (DS 4.5). Accordingly, the mapping for each pharmacophore was performed against a validation set comprising experimentally confirmed BTK inhibitors as actives (isDiverse = 1) and 23,100 ZINC drug-like decoy compounds as decoys (isDiverse = 0). Then, the compounds from the mapping fail file were included in the ROC analysis with the worst-case penalty score and ranked last, as mapping failure constitutes evidence of poor pharmacophoric fit. Then, all the compounds were ranked by ascending Estimate value (RMS fit deviation). In addition to ROC-AUC, the following early-enrichment metrics were calculated for each model: enrichment factor at 1% and 5% of the ranked database (EF1% and EF5%), normalized Boltzmann-enhanced discrimination of ROC (BEDROC, α = 20), sensitivity, and specificity at the optimal Youden index threshold. And the Area Under the Curve (AUC) was calculated for each ROC curve as a quantitative metric of the overall discriminatory performance [32, 39]. Accordingly, these ROC curve complementary metrics jointly address global discrimination (ROC-AUC), early-enrichment performance critical for prospective screening (EF, BEDROC), and classification balance (sensitivity, specificity). Subsequently, the AUC values were compared across the pharmacophore models to determine their relative efficacy in discriminating active BTK inhibitors, with higher AUC values indicating better discriminatory capacity and thus, more robust model performance for virtual screening and lead optimization applications [30–32, 37, 39, 40].

**Table 1:** Pharmacophoric features and corresponding weights, tolerances and 3D coordinates of pharmacophores HypoA-T8-3, HypoE-T8-10, and HypoG-T3-2 that appeared the successful QSAR Eq. (3)

Model	Definition	Chemical features							
GFA-HypoA-T8-10^a^			HBA^h^		HBD^e^		Hbic^f^	Hbic	Hbic
	Weights		2.379		2.38		1.67	2.38	3.81
	Tolerance^d^s^d^		1.3	1.9	1.6	2.2	1.45	1.45	1.45
	Coordinates	X	1.55	0.933	2.85	5.07	1.18	3.74	0.22
		Y	5.10	7.98	− 4.15	− 5.68	− 6.25	2.64	1.11
		Z	3.15	3.77	1.10	− 0.19	− 1.08	0.42	4.59

GFA-HypoE-T8-10^b^			HBA^h^		HBA^e^		Hbic^f^	Hbic	Hbic^g^
	Weights		2.89		2.89		2.89	2.89	3.75
	Tolerances^dd^		1.75	2.2	1.75	2.2	1.6	1.6	1.6
	Coordinates	X	0.158	− 0.162	0.719	0.639	3.56	0.76	0.72
		Y	0.899	− 1.466	3.512	4.605	4.8	− 2.93	3.4
		Z	0.297	2.109	− 3.392	− 6.206	6.36	− 0.417	2.42

GFA-HypoG-T3-2^**c**^			HBA^h^		HBD^e^		Hbic^f^	Pos^g^
	Weights		2.26		2.94		3.61	2.26
	Tolerances^dd^		1.6	2.2	1.6	2.2	1.45	1.45
	Coordinates	X	−6.45	−9.275	0.162	1.567	−3.24	−0.049
		Y	1.079	1.17	3.482	4.478	1.02	−2.357
		**Z**	0.757	1.839	5.906	3.448	1.98	6.007

^a^**Hypo A-T8-10:** the 10th pharmacophore hypothesis generated during the 8th trial on Set A (Table 3 and Table B under Supplementary Materials).

**Hypo A-T8-10** includes 5 exclusion spheres of 1.2 Å diameters and at the following X, Y, Z coordinates: (4.25, −1.34, 6.03), (3.62,-5.99, 1.40), (1.51, −3.45, −2.11),

(−1.83, 3.37, 1.68), (3.57, 3.77, 5.66).

^b^**Hypo E-T8-10:** the 10th pharmacophore hypothesis generated during the 8th trial on Set E (Table 3 and Table B under Supplementary Materials).

**HypoE-T8-10** includes 2 exclusion spheres of 1.2 Å diameters and at the following X, Y, Z coordinates: (-3.80, 3.53, -1.52), (4.50, 8.78, 6.41).

^**c**^**Hypo G-T3-2**: the 2nd pharmacophore hypothesis generated during the 3rd trial on Set G (Table 3 and Table B under Supplementary Materials).

^d^Tolerances: refer to the radius of feature spheres

^e^HBD: Hydrogen Bond Donor, ^f^Hbic: Hydrophobic feature feature, ^g^Pos: Positive ionizable group, ^h^HBA: Hydrogen Bond Acceptor

#### Feature importance analysis, SHAP (shapley additive explanations)

**Fig. 5:**
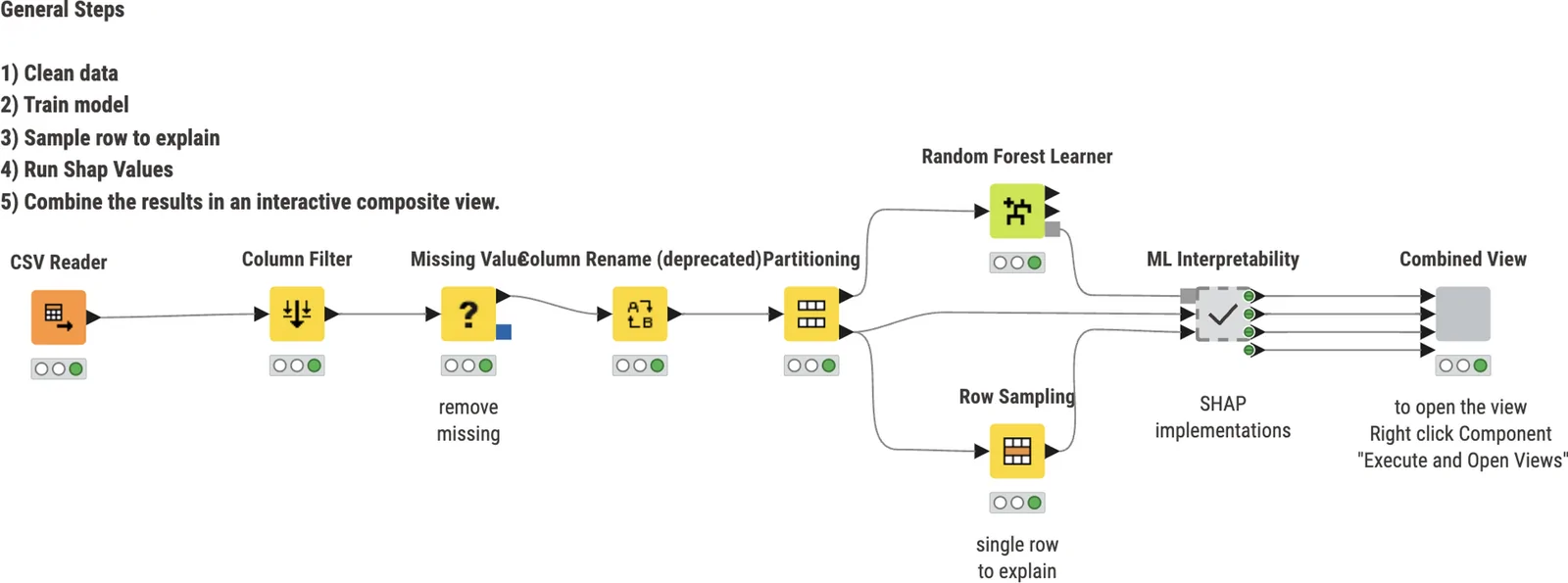
KNIME workflow segment demonstrating SHAP, for calculating the feature importance of BTK inhibitors predictions on a random forest model

The directionality and magnitude of contributions for each molecular descriptor in the successful Forest Tree model in Table 1 were quantified by using SHAP (Shapley Additive Explanations) as visualized in the KNIME workflow segment (Fig. 5). SHAP analysis as demonstrated in Fig. 5 aims to reveal which molecular properties were most influential in determining anti-BTK activity as predicted by the RF model. As such, the “Feature Selection End node” in the SHAP workflow (Fig. 5) iteratively evaluated different subsets of features by guiding the prediction scores from the preceding “scorer node” which effectively refined the feature set.

### Virtual screening and hit selection

GFA-HypoA-T8-10, ML-HypoB-T2-5, and GFA-HypoG-T3-2 (Tables 1 and 2) were used to virtually screen the National Cancer Institute (NCI) diversity database for new active hits against BTK enzyme (Table 3). ML-HypoH-T8-4 was excluded from NCI database screening due to its poor discriminatory performance in the extended ROC validation (ROC-AUC = 0.483, EF1% = 0.91, BEDROC = 0.074, specificity = 34.1%), as detailed in Table 4. GFA-HypoE-T8-10 was also excluded from prospective screening despite showing good overall AUC (0.873), as its lower specificity (72.9%) and EF values (EF1% = 3.28) relative to the three selected pharmacophores made it less suitable for prospective screening where false-positive rate control is critical.

**Table 2:** Comparative performance of machine learning models in predicting BTK inhibitor activity

Model (learner)	Feature set^[a]^	Internal validation^[b]^		External validation^[c]^	
		Accuracy (%)	Cohen’s kappa (κ)	Accuracy (%)	Cohen’s kappa (κ)
RANDOM FOREST	Physicochem., Pharmacophore (Set 1)*	99.28	0.99	94.74	0.92
XGBOOST LINEAR MODEL	Physicochem., Pharmacophore (Set 2)*	96.38	0.94	92.11	0.88
NAIVE BAYES	Physicochem., Pharmacophore (Set 3)*	90.58	0.85	89.47	0.84
K-NEAREST NEIGHBORS	Pharmacophore (Set 4)*	89.85	0.84	89.47	0.84
RPROP MULTILAYER PERCEPTRON	Shape-based, electrostatic (Set 5)*	86.23	0.78	86.84	0.8
SUPPORT VECTOR MACHINES (SVM)	Pharmacophore (Set 4)*	42.75	0	28.95	−0.11

^[a]^**Feature set indicates the molecular and pharmacophoric combination that resulted from each model. By comparing across different feature sets, we can see how the output information affects prediction accuracy**

**Set 1** (Physicochem., Pharmacophore): Dipole Y DMol3, B T2 5, H T8 4, Molecular volume Propgen VAMP, Covalent hydrogen bond acidity Propgen VAMP, Octupole YYZ VAMP, Jurs DPSA3, NumHBD

**Set 2** (Physicochem., Pharmacophore): Dipole Y DMol3, B T2 5, H T8 4, Molecular volume Propgen VAMP, Covalent hydrogen bond acidity Propgen VAMP, Octupole YYZ VAMP, Jurs DPSA3, ExactMW

**Set 3** (Physicochem., Pharmacophore): Dipole Y DMol3, B T2 5, H T8 6, Molecular surface area Propgen VAMP, Covalent hydrogen bond basicity Propgen VAMP, Octupole YZZ VAMP, Jurs FNSA 1, NumHBA

**Set 4** (Pharmacophore): B T5 4, B T7 5, F T3 7, H T1 6, H T1 9, Num RotatableBonds, Jurs FNSA 3, Jurs RPCG

**Set 5** (Shape-Based, Electrostatic): B T5 6, F T4 10, H T1 9, ESP point count 5 Propgen VAMP, RIJestateSumI Propgen VAMP, H Count, Dipole mag, Shadow XY

[b] Internal Validation: Performance metrics derived from cross-validation on the training dataset

[c] External Validation: Performance metrics evaluated on an independent external test dataset

**Table 3:** Pharmacophoric features and corresponding weights, tolerances and 3D coordinates of pharmacophores HypoB-T2-5, and HypoH-T8-4 that appeared in the successful machine learning models in Table 1

Model	Definition	Chemical features							
ML-HypoB-T2-5^a^			HBA^d^		HBA^d^		Hbic^e^	Hbic^e^	Pos^f^
	Weights		3.11		2.62		1.15	1.15	1.15
	Tolerance^c^		1.3	1.9	1.6	2.2	1.3	1.75	1.75
	Coordinates	X	−5.1	−5.67	−4.93	−2.06	−2.24	−6.82	3.48
		Y	1.1	3.93	−3.02	−3.79	1.75	−3	4.02
		Z	1.13	0.33	1.62	2.08	1.64	1.74	0.76

ML-HypoH-T8-4^b^	Definition		HBA^d^		HBA^d^		Hbic^e^	Hbic^e^	Hbic^e^
	Weights		1.09		2.02		1.09	2.48	2.95
	Tolerances^c^		1.6	1.9	1.75	1.9	1.3	1.6	1.45
	Coordinates	X	2.48	4.17	− 1.65	− 0.74	− 3.82	3.7	2.88
		Y	− 1.25	− 1.92	− 0.80	0.31	0.44	− 3.06	− 3.72
		Z	− 1.97	− 4.38	− 2.39	− 5.04	0.1	2.02	5.16

^a^Hypo B-T2-5: the 5th pharmacophore hypothesis generated during the 2nd trial on Set B (Table 3 and Table B under Supplementary Materials)

^b^**Hypo H-T8-4:** the 4th pharmacophore hypothesis generated during the 8th trial on Set H (Table 3 and Table B under Supplementary Materials)

HypoH-T8-4 includes 5 exclusion spheres of 1.2 Å diameters and at the following X, Y, Z coordinates: (− 2.94, − 5.47, 3.02), (− 1.67, − 2.48, 9.53), (− 2.02, − 2.62, 3.77),

(7.01, − 4.87, 5.07), (7.2, 0.59, 5.08)

^c^Tolerances: refer to the radius of feature spheres

^d^HBA: Hydrogen Bond Acceptor^, e^Hbic: Hydrophobic feature feature, ^f^Pos: Positive ionizable group,

**Table 4:** ROC curve analysis criteria for QSAR and machine learning selected pharmacophores

Pharmacophore model	ROC-AUC	ROC Eval	EF 1%	EF 5%	BEDROC (α = 20)	Sens. (%)	Spec. (%)	Acc. (%)
*QSAR-GFA pharmacophore models*								
GFA-HypoA-T8-10	0.984	Excellent	23.72	13.04	0.821	100.0	96.4	96.5
GFA-HypoE-T8-10	0.873	Good	3.28	3.35	0.671	85.1	72.9	75.3
GFA-HypoG-T3-2	1.000	Excellent	9.11	9.11	1.000	100.0	100.0	100.0
*Machine learning pharmacophore models*								
ML-HypoB-T2-5	1.000	Excellent	7.88	7.88	1.000	100.0	100.0	100.0
ML-HypoH-T8-4	0.483	Poor	0.91	0.39	0.074	100.0	34.1	42.1

QSAR-GFA Best Pharmacophore Model are extracted from the successful Eq. (3) based on internal and external validation metrics

Machine Learning Best Pharmacophore Model are the pharmacophores that appeared in the successful machine learnning model in Table 1

^a^**ROC**: receiver operating characteristic curve. ^b^**AUC**: area under the ROC curve, quantifying overall discriminatory power across all classification thresholds (0.5 = random; 1.0 = perfect discrimination). ^c^**ROC Evaluation**: qualitative classification of AUC performance (Excellent: AUC ≥ 0.9; Good: 0.75–0.89; Moderate: 0.60–0.74; Poor: < 0.60). ^d^**EF 1% and EF 5%** (Enrichment Factor): the concentration of true active BTK inhibitors within the top 1% and top 5% of the pharmacophore-ranked compound list relative to random selection, calculated as EF(x%) = [Hits(x%) / Top_N(x%)] / [N_actives / N_total]; an EF of 1.0 corresponds to random performance, while higher values indicate superior early enrichment relevant to prospective virtual screening. ^e^**BEDROC** (α = 20): Boltzmann-Enhanced Discrimination of ROC, an early-recognition metric (Truchon & Bayly, J. Chem. Inf. Model. 2007, 47, 488–508) that applies exponential weighting to penalize the late placement of actives in the ranked list, normalized to a [0, 1] scale where 1.0 represents ideal early recognition (all actives ranked first) and 0.0 represents the worst-case ranking. ^f^**Sens.** (Sensitivity, %): the true positive rate, describing the percentage of known active BTK inhibitors correctly identified by the pharmacophore at the optimal Youden index threshold (Sensitivity = TP/(TP + FN)). ^g^**Spec.** (Specificity, %): the true negative rate, describing the percentage of inactive/decoy compounds correctly rejected by the pharmacophore at the optimal Youden index threshold (Specificity = TN/(TN + FP)). ^h^**Acc.** (Accuracy, %): the overall percentage of compounds correctly classified (active or inactive) by the pharmacophore at the optimal Youden index threshold (Accuracy = (TP + TN)/N × 100) [41]

The NCI database screening workflow was implemented in a multi-step manner by applying the pharmacophores GFA-HypoA-T8-10, ML-HypoB-T2-5, and GFA-HypoG-T3-2 (as detailed in Table 4) to screen the NCI diversity database set. As mentioned earlier, the pharmacophoric models GFA-HypoA-T8-10, ML-HypoB-T2-5, and GFA-HypoG-T3-2 were carefully selected to represent the top-performing models from both branch 1 and branch 2. Next, the NCI database compounds were screened for their ability to match the key chemical features as defined by pharmacophores GFA-HypoA-T8-10, ML-HypoB-T2-5, and GFA-HypoG-T3-2, and a pharmacophore fit score was calculated for each compound. Subsequently, to ensure drug-likeness, we applied Lipinski's Rule of 5 and Veber's Rule and the hit selection process proceeded through a multi-criteria approach, integrating the predictions from both the machine learning and pharmacophore screening phases.

### Model prediction methodology

The ML-RF (PCA) predictions were obtained by projecting pharmacophore fit values and selected physicochemical descriptors into a principal component space which was followed by activity estimation using a trained Random Forest model. Subsequently, the principal component equation incorporated key contributors such as H-T8-4 and B-T2-5 pharmacophore fits, hydrogen-bond acidity, dipole moment, molecular volume, polar surface area descriptors, and hydrogen bond donor count, as defined by the RF-PC1 formulation used during model construction prediction.

On the other hand, the GFA-MLR predictions were generated using a QSAR model derived via genetic function approximation, in which predicted IC_50_ values were calculated according to the optimized regression equation (Eq. 3). This model emphasizes interpretable structure activity relationships by directly linking pharmacophore alignment scores to predicted biological activity.

#### Applicability domain analysis

The applicability domain (AD) for both the GFA-MLR and Random Forest (ML) models was assessed by using the leverage approach (Williams’s plot), as described by [42] and [41, 43, 44]. Thus, the warning leverage threshold was calculated as: h* = 3(k + 1)/n, where k is the number of descriptors and n is the number of training compounds. The leverage was computed for the training set, the external test set, and the virtual screening hit compounds, by using the (X^t^X)⁻1 matrix that was derived exclusively from the training set; hit and external test compounds that were projected into this fixed descriptor space without altering the underlying model. As for the GFA-MLR model, the nine descriptors comprising Eq. 3 were used namely, (ALogP, electrostatic hydrogen-bond acidity, Jurs FNSA-1, Jurs WNSA-1, Shadow XY-fraction, total energy, and the GFA-HypoA-T8-10, GFA-HypoE-T8-10, and GFA-HypoG-T3-2 pharmacophoric fit values; k = 9). Also, for the Random Forest model, leverage was computed by using the three continuous physicochemical descriptors among the GA-selected feature set (Dipole Y_DMol3], molecular volume, and covalent hydrogen-bond acidity; k = 3).

### Biological evaluation by using cytotoxicity assays

The cytotoxicity inhibitory activities of 28 NCI compounds were evaluated on CLL Raji Lymphoma and K562 Leukemia cell lines. Upon applying the assay, a total of 40,000 and 30.000 cells were seeded per well for CLL Raji Lymphoma and K562 leukemia, respectively. According to the test protocol, the plates were incubated overnight with 5% CO_2_ at 37 °C and then, six concentrations of each tested NCI compound were added (ranging from 3 to 100 μM). Next, the assay was repeated at lower concentrations for the compounds that showed a % survival rate above 50% at the concentration of 3 µM. The cells were incubated for another 72 h at the same conditions, and their proliferation was investigated by using the MTT cell proliferation assay kit (Promega Corporation, USA), according to the manufacturer's protocol. Finally, the cell % survival was measured at 570 nm by using a microplate reader (Biotek, Winooski, VT, USA), and the IC_50_ values were calculated by using the non-linear regression function on GraphPad Prism version 9.0.0 software (Table 5).

**Table 5:** Virtual screening and experimental validation outcomes

Pharmacophore model	Initial hits (screening)	Passed filtering^†^	Failed filtering	Compounds acquired from NCI^*^	Experimentally active^‡^	Pharmacophore success rate (%)
GFA-HypoA-T8-10	3599	1,961	1638	5	3	60.0
GFA-HypoE-T8-10	57,820	40,273	17,547	15	3	20.0
GFA-HypoG-T3-2	375	207	168	8	2	25.0
ML-HypoB-T2-5	167	77	90	7	2	28.6
ML-HypoH-T8-4	65,492	48,068	17,424	23	7	30.4

^†^Filtering performed using Lipinski’s Rule of Five and Veber’s criteria

^‡^Activity categories based on approximate IC_50_ ranges: Excellent (≤ 10 µM), Low micromolar (10–30 µM), Moderate (30–100 µM), Weak/Inactive (> 100 µM or ND)

*Some compounds may be mutual between two or three pharmacophores

### Statistical correlation analysis between experimentally determined IC_50_ values for active compounds and their corresponding ML-RF and GFA-MLR activity predictions

To evaluate whether our independent prediction frameworks (ML-RF and GFA-MLR) of BTK inhibitor can be translated into experimentally measurable cellular activity we performed a separate correlation analyses for Raji and K562 cell lines by using compounds with experimentally determined IC_50_ values shown in Tables 6 and 7. Consequently, Raji cell correlations included compounds 166, 167, 168, 169, 170, 171, 172, 176, and 177, whereas K562 cell correlations were conducted by using compounds 166, 167, 168, 169, 170, and 171. Then, two independent sets of predicted IC_50_ values were evaluated: (i) predictions generated by using the ML-RF (PCA) model and (ii) predictions obtained from the GFA-MLR QSAR model. Finally, predicted and experimental IC_50_ values were converted to consistent units and log_10_-transformed prior to analysis and the pearson correlation was used to assess linear association, while Spearman rank correlation was employed to evaluate consistency in relative potency ranking.

**Table 6:** Pharmacophore fit values and predicted BTK inhibitory activity from ML-RF (PCA) and GFA-MLR models

Compound	HypoH-T8-4	HypoB-T2-5	HypoA-T8-10	HypoE-T8-10	HypoG-T3-2	Predicted IC_50_ (µM) ML-RF (PCA)	Predicted IC_50_ (µM) GFA-MLR
166	7.63	0.00	8.62	9.64	0.00	0.05–0.10	0.5–1.5
167	8.53	0.00	11.29	13.84	0.00	0.05–0.10	0.1–0.5
168	3.42	6.77	1.73	12.56	8.78	0.05–0.10	3–5
169	5.23	0.00	0.00	13.41	0.00	1–3	5–10
170	N/A	N/A	0.00	2.84	8.48	N/A	1–3
171	N/A	N/A	6.68	14.96	8.52	N/A	0.1–0.5
172	8.13	0.00	9.69	13.88	0.00	0.1–0.3	1–3
173	8.21	0.00	0.00	13.49	0.00	1–3	> 100
174	N/A	N/A	0.00	0.00	10.19	N/A	20–30
176	6.53	0.87	0.00	8.23	8.06	0.3–1	5–10
177	8.35	0.00	0.00	14.67	0.00	50–100	50–100

ML-RF (PCA) predictions are reported as activity ranges reflecting model uncertainty and ranking consistency rather than absolute precision

GFA-MLR predictions are derived from Eq. (3) and expressed as approximate IC_50_ bins to maintain interpretability and consistency with QSAR limitations

N/A indicates compounds outside the ML-RF descriptor applicability domain

**Table 7:** Cytotoxicity of selected compounds against Raji lymphoma and K562 Leukemia cell lines

Compound code	Structure	Raji lymphoma (CLL) cell line	K562 leukemia cell line	Activity category	Notes/observations
166 NCS13003		0.087 µM	0.73 µM	Excellent	Remarkable nanomolar potency in Raji, also potent in K562
167 NCS87905		5.756 µM	8.638 µM	Excellent	Potent against both cell lines
168 NCS172774		4.92 µM	4.37 µM	Excellent	Potent against both cell lines
169 NCS82451		12.73 µM	6.18 µM	Low micromolar	Low micromolar activity in Raji, moderate in K562
170 NCS115429		21.78 µM	11.13 µM	Low micromolar	Moderate activity against both cell lines
171 NCS527453		11.23 µM	4.26 µM	Low micromolar	Moderate activity against both cell lines
172 NCS28563		27.34 µM	9.29 µM	Moderate	Moderate in Raji, Weak/Inactive in K562
173 NCS81488		ND	47.68 µM	Weak/inactive	Not Determined in Raji, Weak/Inactive in K562
174 NCS131330		53.27 µM	ND	Weak/inactive	Weak/Inactive in Raji, Not Determined in K562
175 NCS663626		46.03 µM	104.2 µM	Weak/inactive	Weak/Inactive in both cell lines
176 NCS18213		158.6 µM	ND	Weak/inactive	Inactive in both cell lines at tested conc
177 NCS161068		11.79 µM	ND	Low micromolar	IC50 value in Raji is uncertain, Not Determined in K562

*Activity Categories*: Categories are based on approximate IC50 ranges: Excellent (IC_50_ ≤ 10 µM), Low Micromolar (10 µM < IC50 ≤ 30 µM), Moderate (30 µM < IC_50_ ≤ 100 µM), Weak/Inactive (IC_50_ > 100 µM or ND). ND: Not Determined which indicates no IC_50_ value was determined at tested concentrations

### Evaluation of the acridine based compounds against the BTK enzyme

To assess the inhibitory activity of the acridine based hit compounds 166 and 168 (Table 7) against the BTK enzyme, an in-vitro recombinant human BTK biochemical kinase assay was employed by Reaction Biology Corp. (Malvern, PA, USA) to quantify the ATP phosphorylation of the BTK enzyme in the presence and absence of hit compounds 166 and 168.

Later, the test compounds (166 and 168, Table 7) were dissolved as stock solutions in dimethyl sulfoxide (DMSO) and then serially diluted to obtain concentration profiles that range from 1.0 × 10⁻^4^ M to 5.08 × 10⁻⁹ M. And so, the reactions were performed in assay buffer containing HEPES, MgCl_2_, and a reducing agent under optimized conditions. Prior to initiation of the kinase reaction, the recombinant BTK enzyme was allowed to incubate with each test compound at the indicated concentrations to allow the inhibitor-enzyme interaction. Then, the enzymatic reaction was initiated by the addition of ATP (1 μM) and peptide substrate. Following incubation, kinase activity was quantified by measuring the luminescence intensity proportional to ATP consumption. Moreover, the known kinase inhibitor, Staurosporine, was included as a positive control to validate our assay performance. All measurements were performed in replicate wells to ensure their reproducibility and the dose–response curves were generated. Finally, IC_50_ values were calculated by using the non-linear regression analysis.

### Molecular docking and protocol validation

All docking and molecular dynamics simulations were performed using the Schrödinger 2025-2 [45] suite. Crystal structures of BTK were retrieved from the Protein Data Bank (www.RCSB.org): PDB ID 6X3N (resolution: 1.95 Å), PDB ID 5VFI (resolution: 1.59 Å), and PDB ID 5T18 (resolution: 1.50 Å). Then, each structure was prepared by using the Protein Preparation workflow, which assigns bond orders and pKa values. Specifically, compound 166 was prepared by using the LigPrep module, with maintaining a neutral state of the tertiary amine group based on available structural information from BTK inhibitor complexes that reveal solvent-accessible binding of positively charged moieties. This constraint was applied to minimize the risk of introducing artificial electrostatic interactions that could bias docking poses. OPLS4 force-field dihedral parameters for compound 166 were evaluated and parameterized by using the Force Field Builder tool.

The Induced Fit Docking (IFD) was performed to investigate the potential binding pose of compound 166 within the BTK active site and to rationalize its ability to inhibit the T474I mutant. The IFD protocol maintained conformational flexibility of both the ligand and protein, initiated with Glide docking at 50% van der Waals radii downsizing for protein and ligand, followed by Prime minimization and optimization of residues within 5 Å of the ligand binding pose.

Three BTK crystal structures were selected for IFD, each representing a distinct co-crystallized inhibitor binding mode within the ATP-binding site: (1) PDB ID 6X3N, in which the co-crystallized inhibitor is positioned below the catalytic lysine (K430) and co-occupies the hydrophobic back pocket (M449, I472, and L542); (2) PDB ID 5VFI, in which the co-crystallized ligand is positioned above K430 and co-occupies the H3 pocket (Q412, F413, L542, S543, and Y551); and (3) PDB ID 5T18, in which the co-crystallized ligand (BMS-986142) does not engage in hydrogen bond interaction with K430 while occupying only the hinge region. Structural analysis of available inhibitor-bound BTK crystal structures indicates that all binding modes share a common hydrogen bond interaction with the hinge region residue M477, and that these different modes influence the conformational state of full-length BTK, thereby affecting its activity in B-cell receptor signaling.

To validate the docking protocol, the co-crystallized ligand was removed from the BTK crystal structure (PDB ID 5T18) and re-docked into the ATP-binding site using the same IFD parameters applied to compound 166. The re-docked pose reproduced the crystallographic binding orientation with an RMSD of 0.83 Å (Fig. S1), which is well within the accepted threshold of < 2.0 Å, confirming the reliability of the docking protocol. The validated protocol was subsequently employed for docking compound 166 into all three receptor conformations.

Based on structural comparison with co-crystallized inhibitors, we hypothesized that compound 166 would preferentially adopt a hinge-region-only binding mode, as observed in the crystal structures of BTK in complex with BMS-986142 (PDB ID: 5T18) and BMS-935177 (PDB ID: 5JRS).

### Molecular dynamics simulations

Classical molecular dynamics (MD) simulations were performed using the Desmond simulation package implemented in the Schrödinger Suite. The BTK–compound 166 complex was embedded in an explicit cubic water box using the TIP3P solvent model and neutralized with appropriate counter ions. After system relaxation and equilibration under NVT and NPT ensembles, production MD simulations of 100 ns were carried out under periodic boundary conditions at 310 K and 1 atm using the optimized OPLS4 force field. Three representative docking poses of compound 166 were selected for MD evaluation based on their ability to form the essential hydrogen bond interaction with hinge region residue M477, and each pose was simulated in duplicate (six simulations total, 600 ns cumulative). Trajectory analyses were performed using the Simulation Interaction Diagram module to evaluate ligand binding pose stability, protein–ligand interaction persistence, hydrogen bond occupancy, and root-mean-square deviation (RMSD) and fluctuation (RMSF) profiles.

### Novelty assessment of hits

To achieve a novelty proof for our captured anti-BTK new hits a Principal Component Analysis (PCA) study was employed using the DS 4.5 protocol that can show the relative distribution of captured active hits in Table 6 when compared to modeled compounds having IC_50_ ≤ 9 nM. And so, our diversity analysis was performed using a predefined set of molecular properties including the ALogP, Molecular weight, number of HBD, number of HBA, number of rotable bonds, number of aliphatic and aromatic rings, molecular surface area, molecular polar surface area, number of atoms, and number of fragments.

Furthermore, to complement the PCA diversity study with a direct, quantitative test of structural novelty, a library comparison analysis was performed by using the “Compare Libraries” protocol in the DS 4.5. So, the full set of the 165 training and test compounds (Library A) was compared against the captured active hit compounds (Table 6; Library B) by using three independent comparison methods: (i) The Bemis–Murcko scaffold (Assemblies) analysis, which fragments each ligand into its core scaffold and computes a Tanimoto similarity coefficient based on scaffolds common to and unique to each library; (ii) The Bayesian model-based distance metric, in which independent Bayesian models were trained to learn each library and then used to cross-score both libraries, with the distance computed as the sum of the average score of Library A molecules scored by the Library A model and the average score of Library B molecules scored by the Library B model, minus the average cross-scores (Library A molecules scored by the Library B model, and Library B molecules scored by the Library A model), such that larger distance values indicate greater dissimilarity between the libraries; and (iii) The global molecular fingerprint comparison using the FCFC_6 fingerprint, in which a global fingerprint is generated for all ligands in each library and a Tanimoto similarity coefficient is computed between the two fingerprint sets.

## Results

Our integrated computational workflow (see Fig. 2) was able to successfully combine ligand-based pharmacophore modeling, QSAR-GFA analysis, and machine learning classification to identify novel BTK inhibitors. The curated dataset of 165 structurally diverse BTK inhibitors (see Table A in supplementary material) was used for model development and validation, this was followed by virtual screening of the NCI diversity database and experimental validation via cytotoxicity assays (Figs. 2 and 3).

### Results of branch 1: discovery studio (DS) QSAR-GFA model

Branch 1 of this study focused on developing a quantitative structure–activity relationship (QSAR) model by using Genetic Function Approximation and Multiple Linear Regression (GFA-MLR) within Discovery Studio 4.5 [46]. Hence, the resulting GFA-MLR equation, derived from the Discovery Studio workflow for predicting BTK Log(1/IC_50_) activity is:

3 $$ \begin{aligned} {\mathrm{Log}}\left( {1/{\mathrm{IC}}_{{50}} } \right) = & - 11.46 - 0.15082*\left( {{\mathbf{ALogP}}} \right) \\ & + 14.807*\left( {{\mathbf{Electrostatic}}{\mkern 1mu} {\mathbf{hydrogen}}{\mkern 1mu} {\mathbf{bond}}{\mkern 1mu} {\mathbf{acidity}}} \right) \\ & + 16.268*\left( {{\mathbf{Jurs}}{\mkern 1mu} {\mathbf{FNSA}} - {\mathbf{1}}} \right) \\ & - 0.026795*\left( {{\mathbf{Jurs}}{\mkern 1mu} {\mathbf{WNSA}} - {\mathbf{1}}} \right) \\ & - 4.6361*\left( {{\mathbf{Shadow}}{\mkern 1mu} {\mathbf{XYfraction}}} \right) \\ & - 0.0013336*\left( {{\mathbf{Total}}{\mkern 1mu} {\mathbf{Energy}}} \right) \\ & + 0.14428*\left( {{\mathbf{HypoA}} - {\mathbf{T8}} - {\mathbf{10}}} \right) \\ & + 0.094977*\left( {{\mathbf{HypoE}} - {\mathbf{T8}} - {\mathbf{1}}0} \right) \\ & + 0.13698*({\mathbf{HYpoG}} - {\mathbf{T3}} - {\mathbf{2}}) \\ \end{aligned} $$

#### Validation parameters using internal test

N = 127, r^2^_127_ = 0.60, r^2^_adj_ = 0.56, Friedman L.O.F. = 2.96, *p*-value = 4.93 e-019, r^2^_pred_ = 0.51.

#### Validation parameters using external test

N_external_ = 38, Q^2^ = 0.55, RMS Error = 0.98, Mean Absolute Error = 0.74.

This successful GFA-MLR model (Eq. (3)) can quantitatively describe the contribution of nine important independent variables to BTK inhibitory activity which includes descriptors that are capturing complementary physicochemical, spatial, and pharmacophoric aspects of ligand interactions inside the BTK binding pocket. Specifically, ALogP which represents molecular lipophilicity, while electrostatic hydrogen bond acidity reflects the electrostatic contribution of hydrogen bond donating functionalities. Moreover, the Jurs descriptors (FNSA-1 and WNSA-1) are describing the fractional and weighted negatively charged molecular surface areas, providing insight into surface electrostatics relevant to BTK kinase binding. Furthermore, shadow XYfraction encodes molecular shape and projected size in the XY plane, and total Energy represents the optimized molecular energy calculated using the VAMP method [31, 33].

In addition to classical physicochemical descriptors, three pharmacophore fit values (HypoA-T8-10, HypoE-T8-10, and HypoG-T3-2) (see Table 1) are incorporated into the successful model (Eq. 3). Thus, the pharmacophoric fit values quantify the degree of geometric alignment between each compound in the training set and the corresponding pharmacophore hypotheses derived within Branch 1. And so, the pharmacophoric features, weights, tolerances, and three-dimensional coordinates for these hypotheses are summarized in Table 1.

Also, by referring to Eq. (3) the reader can see that among the included descriptors is the electrostatic hydrogen bond acidity which exhibits a strong positive coefficient (+ 14.807), and this indicates that this descriptor contributes substantially to increasing predicted BTK inhibitory potency. And so, the magnitude of this coefficient, which exceeds the absolute value of the model intercept, highlights the dominant role of electrostatic hydrogen bond donor character in modulating BTK activity within the modeled dataset. Similarly, Jurs FNSA-1 displays a large positive coefficient (+ 16.268) which reflects the importance of negatively charged surface area in promoting favorable interactions with the electron deficient areas inside the BTK binding environment.

Moreover, the strong positive coefficient that is associated with the electrostatic hydrogen bond acidity (+ 14.807) in Eq. (3) aligns well with the prominent hydrogen bond donor interactions observed in Fig. 1 such as the interactions with the charged amino acids Lys430 and Asp539. And so, these interactions emphasize the importance of hydrogen bond donor capability for stabilizing ligand–BTK complexes. Similarly, the large positive contribution of Jurs FNSA-1 (+ 16.268) reflects the relevance of the electron rich and negatively charged molecular surface areas, which complement the electron deficient regions of the BTK binding pocket highlighted in Fig. 1.

**Fig. 6:**
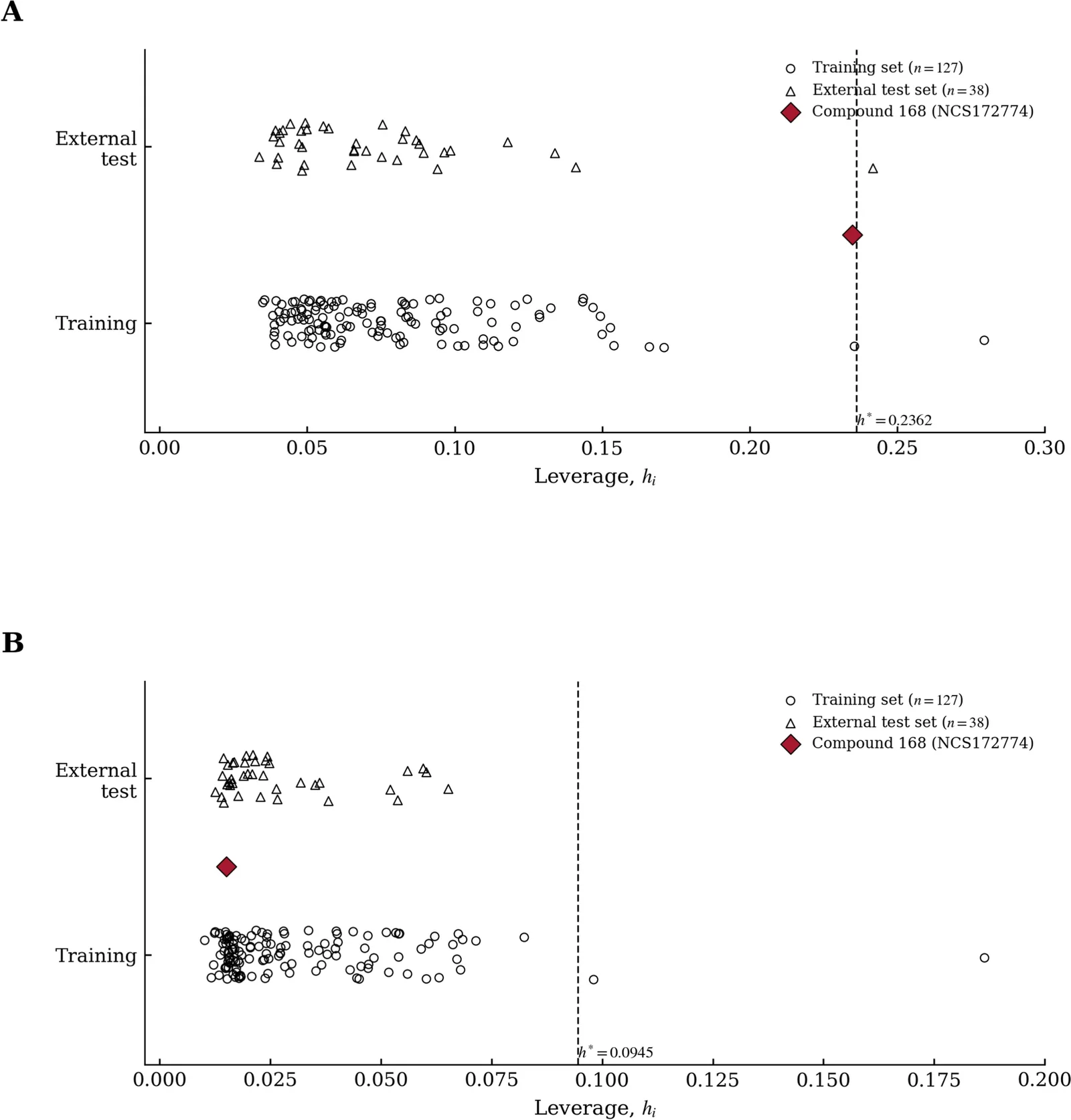
Applicability domain (AD) of the **A** GFA-MLR and **B** Random Forest (ML) models, assessed by leverage (Williams plot approach). Open circles and triangles denote training (n = 127) and external test (n = 38) compounds, respectively; the dashed line marks the warning leverage threshold h* = 3(k + 1)/n. The diamond marks compound 168 (NCS172774), one of two hit compounds advanced to direct BTK enzyme inhibition assay. Compound 168 falls within the AD of both models (h = 0.235 vs. h* = 0.236 for GFA-MLR; h = 0.015 vs. h* = 0.095 for the Random Forest model)

On the other hand, the descriptors that are related to excessive molecular size and shape projection, including ALogP and Shadow XYfraction are displaying negative coefficients (−0.15082 and −4.6361) which suggests a negative effect on the predicted IC_50_ inhibitory values. Furthermore, the negative coefficients for ALogP and Shadow XYfraction are consistent with Fig. 1, where effective BTK inhibitors adopt compact and well-oriented binding poses rather than excessively lipophilic or laterally extended conformations since this would disrupt the precise positioning that is required for productive hinge and electrostatic interactions (Fig. 6).

Interestingly, the reader can also notice the appearance of pharmacophore fit values (HypoA-T8-10 (+ 0.14428), HypoE-T8-10 (+ 0.094977), and HypoG-T3-2 (+ 0.13698)) with consistently positive coefficients which confirms that improved geometric and chemical alignment with the derived pharmacophore hypotheses contributes positively to BTK inhibition. Thus, these terms encode essential 3D binding requirements and provide an explicit structural component to the resulted QSAR model un Eq. (3) (see Table 1).

### Results of branch 2: machine learning model performance

The results of our selected machine learning models feature selection are summarized is Table 2. By referring to Table 2 the reader can see that it presents the Cohen's Kappa values for each machine learning model and the sets of features selected for each machine learning model in our study. Overall, machine learning models demonstrated good to excellent predictive performance for BTK inhibitor activity in cross-validation, as indicated by the Cohen's Kappa values. Thus, the Cohen's Kappa values ranged from 0.781 to 0.99 for cross-validation which suggests a substantial level of agreement between the predicted and actual bioactivity for most models, although some models on the external test set are showing less robust performance. Interestingly, the Random Forest algorithm achieved the highest performance, with a Cohen's Kappa of 0.99 in cross-validation and 0.92 over the external testing set. Therefore, this top score clearly indicates that the Random Forest model exhibited the strongest predictive capability for BTK inhibitor activity within our dataset and that it can be used for good generalization to an external set. Meanwhile, the XGBoost linear model also demonstrated strong performance by achieving a Cohen's Kappa values of 0.94 in cross validation and 0.88 on external testing. Next, the RProp Multilayer Perceptron (MLP) showed good performance with Cohen's Kappa values of 0.781 in cross validation and 0.80 in external testing. Finally, Naive Bayes and k-Nearest Neighbors showed moderate predictive performance in both sets, with Cohen’s Kappa values of 0.85 and 0.838 in cross-validation, and 0.84 for both in external testing respectively. In contrast, the Support Vector Machine (SVM) model expressed a highly variable performance (see Table 2) and this contrast suggests a failure to generalize beyond chance for the SVM model with the chosen feature subset. In conclusion, the results clearly demonstrate that ensemble methods such as Random Forest and XGBoost Linear Model, along with MLP, consistently provides a robust and good performing model for predicting BTK inhibitor activity.

### Feature selection results

By referring to the results in Table 2 a clear observation derived is the consistency in the features selected by the Knime Genetic Algorithm (GA) across some high performing models, particularly ensemble methods (Random Forest, XGBoost Linear Model) and to some extent probabilistic methods (Naive Bayes). While not universally consistent across all models listed including distance-based methods (k-Nearest Neighbors), neural networks (RProp Multilayer Perceptron), and Support Vector Machines (SVM). Thus, the GA identified some recurring molecular descriptors as important for predicting BTK inhibitor activity within the top performing models. Specifically, the features 'Dipole Y DMol3' and the pharmacophoric descriptor 'HypoB-T2-5' were selected by the GA for the Random Forest (Accuracy% = 99.28, Cohen’s Kappa = 0.99), XGBoost Linear Model (Accuracy% = 96.38, Cohen’s Kappa = 0.94), and Naive Bayes models (Accuracy% = 90.58, Cohen’s Kappa = 0.85) (Please refer to Table 3 which encompass the machine learning pharmacophoric hypothesis HypoB-T2-5, and HypoH-T8-4 and their corresponding weights, tolerances and 3D coordinates). And so, the pharmacophore ‘Hypo H-T8-4’ is shared between the best two models in Table 1, i.e., Random Forest and XGBoost Linear Models, moreover Naive Bayes selected ‘H-T8-6’, which indicates a potential preference for this pharmacophore. Furthermore, the descriptors “Molecular-volume-Propgen-VAMP” and “Covalent-hydrogen-bond-acidity-Propgen-VAMP” were also frequently selected.

Thus, the recurrent GA selection of descriptors related to dipole moment, molecular volume, and hydrogen bond acidity in the highest performing models underscores the robustness of these feature types as predictors of anti-BTK activity.

As seen in Table 2 it’s important to note that other models, such as k-Nearest Neighbors, RProp Multilayer Perceptron, and SVM, selected different sets of features which suggests that diverse feature combinations can also achieve reasonable, or as in the case of the SVM model, variable, predictive performance that depends on the algorithm and feature subset.

To assess the statistical significance of performance differences among classifiers, the Cohen’s κ distributions generated during GA feature selection were analyzed across five models. The GA optimization evaluated between 2552 (k-NN) and 20,231 (Random Forest) candidate feature subsets per classifier, with each subset scored via tenfold stratified cross-validation, providing a robust distribution of performance estimates. Random Forest achieved the highest mean κ (0.985 ± 0.007; 95% CI [0.9849, 0.9851]; n = 20,231 subsets), followed closely by XGBoost (0.981 ± 0.022; 95% CI [0.9807, 0.9813]; n = 16,425 subsets). Although the difference was statistically significant (Welch’s t = 22.40, *p* < 0.001), the effect size was small (Cohen’s d = 0.245, Δκ = 0.004), indicating comparable predictive accuracy. The key advantage of Random Forest was its 3.1-fold lower variance (SD = 0.007 vs. 0.022), demonstrating superior robustness to feature selection choices; its minimum κ across all evaluated subsets (0.942) remained above the 0.80 threshold for substantial inter-rater agreement. k-Nearest Neighbor and Naive Bayes formed a second performance tier (mean κ = 0.867 and 0.860, respectively), with substantially larger effect sizes relative to Random Forest (Cohen’s d = 1.03 and 1.31, respectively). RProp MLP showed moderate but highly variable performance (mean κ = 0.756 ± 0.202), with some feature subsets producing κ values below zero, indicating predictions worse than chance for certain descriptor combinations.

### Feature importance analysis

**Fig. 7:**
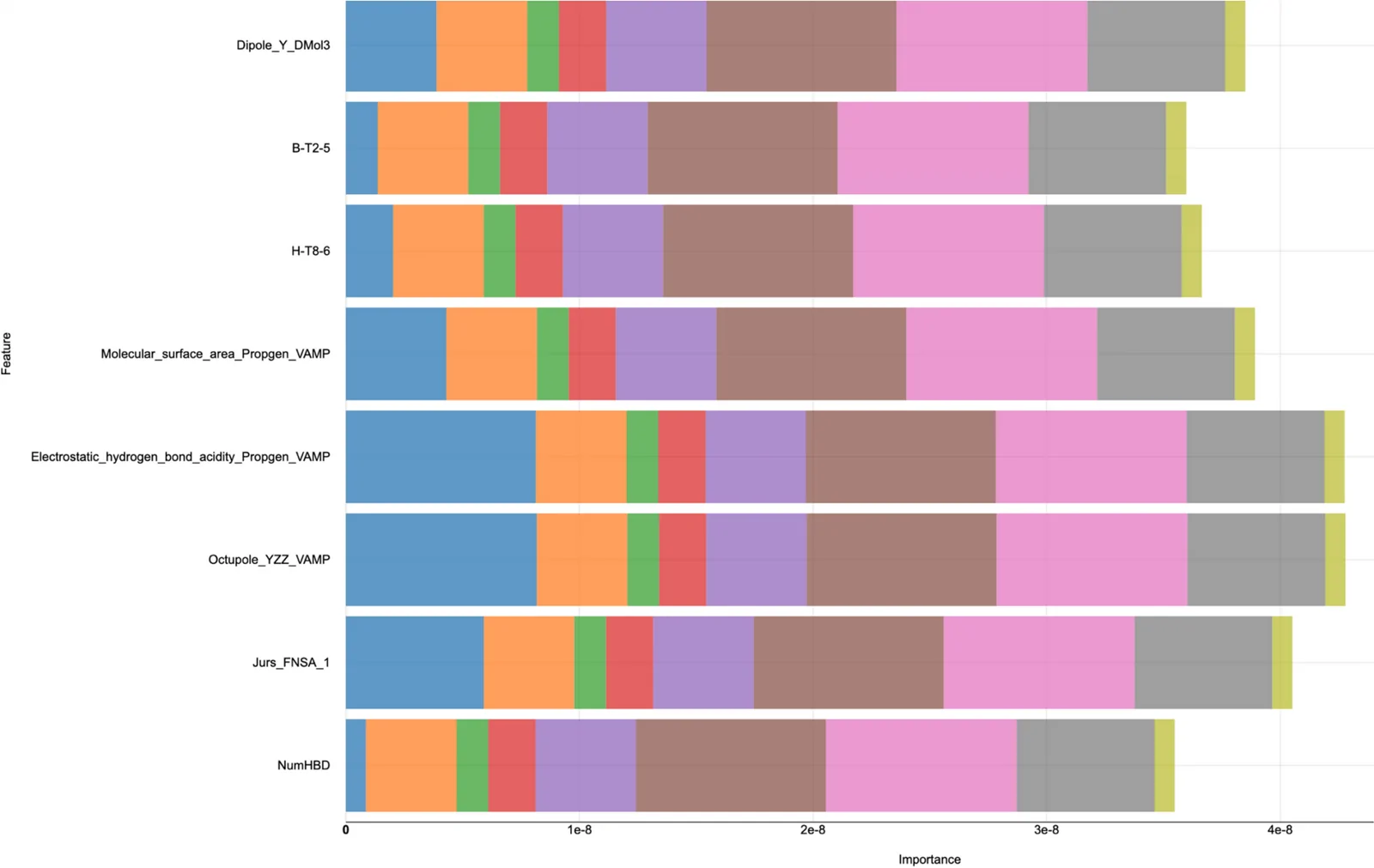
SHAP (SHapley Additive exPlanations) Summary Plot of Feature Importance for BTK Inhibitor Activity Prediction from Branch 2 Machine Learning Random Forest Model. Each row represents a molecular descriptor, ranked by importance from top to bottom. The horizontal axis shows the SHAP value indicates the magnitude and direction of each feature's effect on the RF predicted BTK inhibitor activity

To further investigate feature importance and understand of model predictions pattern, SHAP (SHapley Additive exPlanations) analysis was performed on Random Forest) model (Accuracy% = 99.28, Cohen’s Kappa = 0.99. Thus, a SHAP summary plot (Fig. 7) was generated to visualize the impact of different molecular descriptors on the model's predictions of BTK inhibition. Our SHAP analysis revealed a clear hierarchy of feature importance: Dipole moment along the Y-axis (Dipole-Y-DMol3) and pharmacophores HypoB-T2-5, and HypoH-T8-4 fit values consistently exhibited the highest influence on model predictions. Meanwhile, molecular surface area (Molecular surface area-Propgen-VAMP) showed substantial impact, while electrostatic properties related to hydrogen bond acidity had a more moderate influence. Finally, this hierarchy suggests that while overall molecular properties contribute to BTK inhibition prediction, specific spatial and electrostatic interactions, as captured by pharmacophore features and dipole moment, are paramount.

### Pharmacophore performance evaluation of branch 1 and branch 2 selected pharmacophores via ROC analysis

The results of the ROC validation for all five pharmacophoric models in Tables 1 and 3 are presented in Table 4. Notably, GFA-HypoG-T3-2 and ML-HypoB-T2-5 achieved the perfect discrimination showing the following values: (ROC-AUC = 1.000, EF1% = 9.11 and 7.88 respectively, BEDROC = 1.000, sensitivity = 100.0%, specificity = 100.0%). Also, GFA-HypoA-T8-10 demonstrated excellent performance showing the following values: (ROC-AUC = 0.984, EF1% = 23.72, EF5% = 13.04, BEDROC = 0.821, sensitivity = 100.0%, specificity = 96.4%), with an EF1% of 23.72 indicating nearly 24-fold enrichment of BTK actives in the top 1% of the ranked database relative to random selection. Moreover, GFA-HypoE-T8-10 showed good discriminatory performance (ROC-AUC = 0.873, EF1% = 3.28, EF5% = 3.35, BEDROC = 0.671, sensitivity = 85.1%, specificity = 72.9%), which confirms the meaningful discriminatory ability for the BTK active chemotype. In contrast, ML-HypoH-T8-4 was confirmed as a poor-performing pharmacophore (ROC-AUC = 0.483, EF1% = 0.91, EF5% = 0.39, BEDROC = 0.074, specificity = 34.1%), with below-random AUC and sub-unity EF values which indicates its failure to discriminate BTK actives from ZINC drug-like decoys. Accordingly, GFA-HypoA-T8-10, GFA-HypoG-T3-2, and ML-HypoB-T2-5 were selected for prospective virtual screening of the NCI diversity database, while GFA-HypoE-T8-10 was retained as a supporting model and ML-HypoH-T8-4 was excluded.

### Virtual screening for novel BTK inhibitors

The Branch 1 QSAR-GFA successful model (Eq. 3) and the Random Forest successful machine learning model (see Table 2) were used to virtually screen the NCI database aiming for identifying and prioritizing a set of new anti-BTK active hit molecules. And so, a total of 28 compounds were selected based on their predicted BTK inhibitory activity and pharmacophore fit scores (see Table 5 which describes the progressive virtual screening, predictive prioritization, and experimental validation of candidate BTK inhibitors).

Moreover, Table 6 summarizes the predicted anti-BTK activities for hit compounds 166–177. The predicted activities were generated by using the ML-RF (PCA) and GFA-MLR models. As shown, the predicted IC_50_ values spanned through the sub-micromolar to low-micromolar range plus compounds 166 and 167 were predicted by the ML-RF (PCA) model to fall within the sub-micromolar activity range (0.05–0.10 µM), meanwhile the GFA-MLR model placed these compounds within the 0.1–1.5 µM range which indicates strong predicted potency by both models. As for compound 168, it was predicted to exhibit sub-micromolar activity by the ML-RF (PCA) model, whereas the GFA-MLR model classified it within the low-micromolar range (3–5 µM).

Also, compounds 169, 170, and 172 were predicted to display a low-micromolar activity, with ML-RF (PCA) predictions ranging from 0.1 to 3 µM and GFA-MLR predictions clustering between 1 and 10 µM. In addition, compound 171 was predicted by the GFA-MLR model to fall within the sub-micromolar to low-micromolar range, while ML-RF (PCA) predictions were not reported due to applicability domain constraints. In contrast, compounds 173 and 177 were classified as weak candidates with predicted IC_50_ values exceeding 50 µM.

To assess whether these predictions fall within the reliable interpolation space of each model, an applicability domain (AD) analysis was performed (Section “Applicability Domain Analysis”; Fig. 6). Both models adequately represent their training and external test sets, with leverage values for most compounds in each set falling below the warning threshold h*: 126/127 training and 37/38 external test compounds for the GFA-MLR model, and 125/127 training and 38/38 external test compounds for the Random Forest model (Fig. 6A–B). Among the hit compounds advanced to direct biochemical BTK enzyme inhibition assay, compound 168 lies within the applicability domain of both models (GFA-MLR: h = 0.235, just inside h* = 0.236; Random Forest: h = 0.015, well inside h* = 0.095; Fig. 6), which supports the confidence in its computational prioritization. Compounds 169 and 170 likewise fall within the GFA-MLR domain. In contrast, compounds 166, 167, and 171 exceed the GFA-MLR domain, primarily due to GFA-HypoA-T8-10 or GFA-HypoE-T8-10 pharmacophore fit values exceeding the maximum observed among training compounds. This reflects the fact that these compounds were prioritized by virtual screening specifically for strong pharmacophoric alignment, a property markedly less prevalent among the literature-derived training compounds, rather than indicating general chemical dissimilarity; all three compounds remain within the Random Forest model’s domain on the continuous physicochemical descriptors.

All in all, the results in Table 6 demonstrate that both the ML-RF (PCA) and GFA-MLR models prioritized a subset of compounds with convergent predicted activity in the sub-micromolar to low-micromolar range which supports their selection for subsequent experimental evaluation.

Finally, the experimental validation of the selected anti-BTK hits is reported in Table 7 which lists the NCI codes for the hit compounds with their corresponding experimentally determined IC_50_ values as measured in CLL Raji lymphoma and K562 leukemia cell lines. As shown in Table 7, the observed IC_50_ values spanned from low nanomolar (~ 80 nM) to low micromolar (~ 0.5–5 µM) ranges across both cellular models. Remarkably, most of the hit compounds which are predicted to exhibit nanomolar or sub-micromolar potency in silico demonstrated enhanced antiproliferative activity in vitro which enables direct quantitative comparison between predicted and experimental outcomes. Moreover, the observed variability in cellular IC_50_ values may reflect compound specific ADME related factors which can influence effective intracellular exposure. (the experimentally determined IC_50_ values are summarized in Table 7 and their corresponding dose–response curves are presented in supplementary figure B).

Among the evaluated anti-BTK hits, 166 (NCS13003) which exhibited the highest potency, with an IC_50_ value of 0.087 µM in Raji cells and 0.73 µM in K562 cells. Additionally, another highly active compounds included 167 (NCS87905) with an IC_50_ values of 5.756 µM (Raji) and 8.638 µM (K562), and 168 (NCS172774) with IC_50_ values of 4.92 µM (Raji) and 4.37 µM (K562).

Furthermore, a group of compounds displayed a low-micromolar activity including compound 169 (NCS82451) with IC_50_ values of 12.73 µM (Raji) and 6.18 µM (K562), compound 170 (NCS115429) with 21.78 µM (Raji) and 11.13 µM (K562), and compound 171 (NCS527453) with 11.23 µM (Raji) and 4.26 µM (K562). In addition, four compounds (172, 173, 170, and 171) exhibited moderate cytotoxic activity. Thus, these results confirm the successful experimental validation of our computationally prioritized hits. Finally, the application of this dual-branch virtual screening strategy against the NCI database (255,070 compounds) resulted in distinct hit profiles across GFA-derived and machine learning–derived hypotheses (see Table 5 for the virtual screening and experimental validation outcomes).

### Results correlation between predicted and experimental activity

**Fig. 8:**
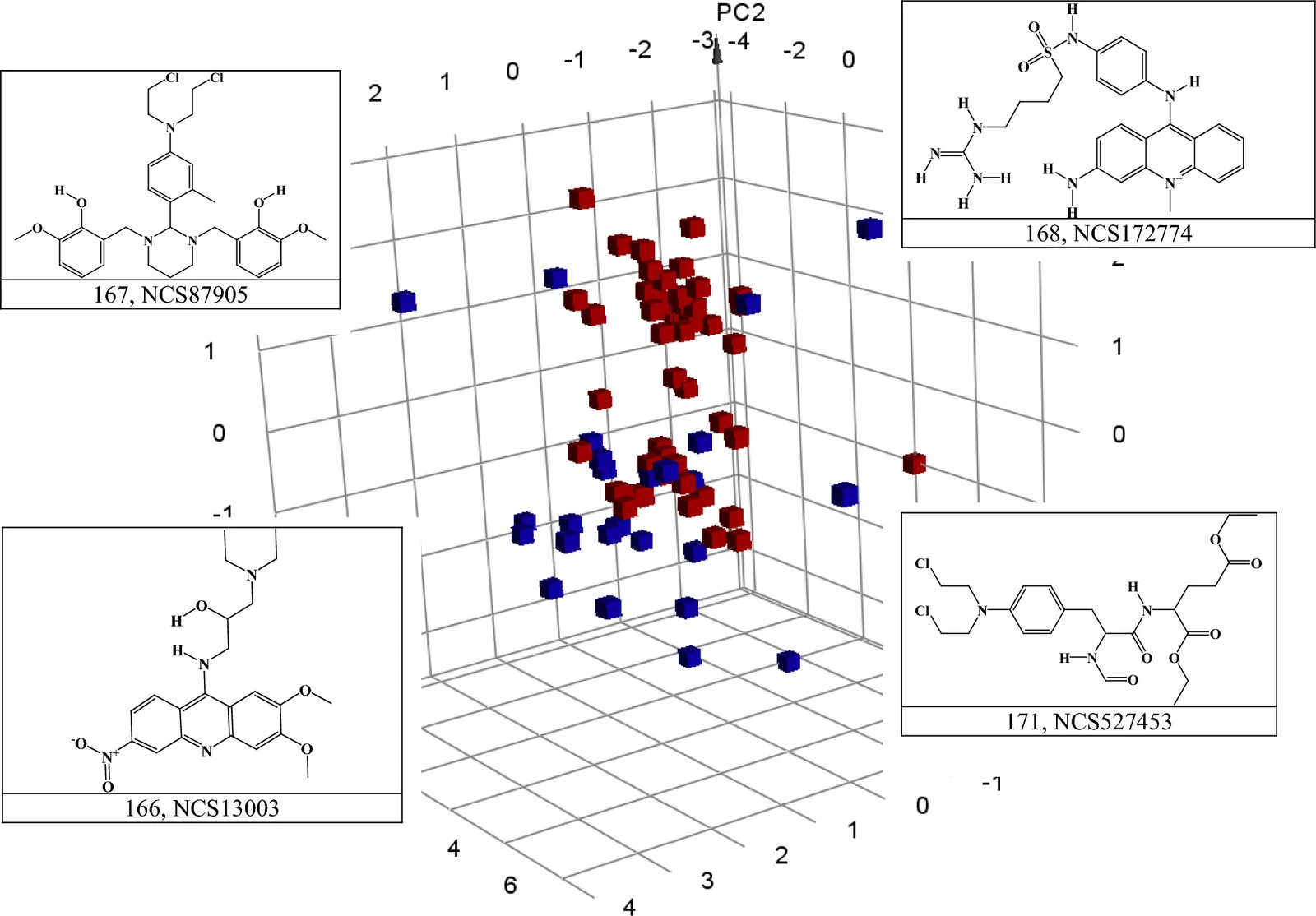
Diversity Study results: Principal Component Analysis showing the relative distribution of captured active hits (blue squares) compared to modeled compounds (red squares), including the original BTK inhibitors of IC_50_ ≤ 9 nM. The top three principal components calculated for modeled compounds and captured hits are based on seven descriptors (i.e., log(P), hydrogen bond donors and acceptors, number of aromatic rings, molecular surface area, molecular polar surface area and molecular fractional polar surface area)

A statistical correlation analysis was conducted to evaluate the agreement between predicted and experimental anti-BTK activities by using two our both independent prediction frameworks (the ML-RF (PCA) and the GFA-MLR frameworks). Thus, the study was first conducted by using log_10_ transformed IC_50_ values, and the analysis results in Raji cells demonstrated moderate positive correlations for both models (see Figure C under supplementary material). Also, the ML-RF (PCA) model showed a Pearson correlation coefficient of *r* = 0.57 (*p* = 0.055) and a Spearman rank correlation of ρ = 0.57 (*p* = 0.051), while the GFA-MLR model exhibited a comparable linear association (*r* = 0.58, *p* = 0.051) and a strong rank order agreement (ρ = 0.62, *p* = 0.031). So, these results indicate consistent preservation of relative potency trends in Raji cells across both models (Fig. 8).

Consequently, the comparison of the experimentally determined IC_50_ values against the ML predicted activities revealed overall agreement in potency trends. For example, the compounds that were predicted to exhibit the highest potency such as compounds 166 and 168, demonstrated the lowest experimental IC_50_ values (~ 80 nM). Furthermore, compounds such as 169, 170, and 171 which showed low micromolar activity (~ 0.5–5 µM, Table 7) also showed consistent predicted activity classifications. Likewise, correlation analysis indicated preserved ranking consistency between predicted and experimental activities, although discrepancies in absolute IC_50_ values were observed in some cases which contributed to the reduced Pearson correlation while maintaining relative ranking. Overall, the experimental data results confirm that the ML-based framework effectively prioritized biologically active compounds spanning sub-micromolar to low-micromolar potency ranges. In contrast, the correlation results in K562 leukemia cells were weaker for both models, which might reflect cell-line-specific ADME factorization or downstream cytotoxic mechanisms.

Overall, this statistical correlation analysis confirms that our applied integrated dual-branch computational strategy effectively prioritizes biologically active cells.

### Evaluation of acridine based compounds against the BTK enzyme

Among the tested compounds, Acridine based compounds 166 and 168 (Table 7) demonstrated measurable inhibition of BTK enzyme, yielding IC_50_ values of 12.2 µM (1.22 × 10⁻^5^ M) and 23.8 µM (2.38 × 10⁻^5^ M), respectively. The reference inhibitor Staurosporine exhibited an IC_50_ value of 11.6 nM (1.16 × 10⁻⁸ M), consistent with its established nanomolar potency and confirming assay validity. Collectively, these findings indicate that compounds 166 and 168 do certainly show inhibitory activity against the BTK enzyme which supports the efficacy of our integrated computational and experimental strategy for the discovery of novel BTK inhibitors.

It is important to note that the BTK enzyme IC_50_ values of compounds 166 and 168 (12.2 and 23.8 µM, respectively) are considerably weaker than their cellular potencies in Raji cells (0.087 and 4.92 µM, respectively), which represents a discrepancy of approximately two orders of magnitude for compound 166. Thus, this divergence indicates that the BTK enzyme inhibition alone does not fully account for the observed antiproliferative activity, and that the cellular effects of compound 166 are most likely to be multi-mechanistic in nature. In this essence, we propose three non-mutual explanations to be considered below. First, the ADME-related factors may partly contribute to this difference. Accordingly, the in-silico ADMET profiling of compounds 166 and 168 was performed by using the “ADMET Descriptors” protocol in BIOVIA Discovery Studio 4.5. And compound 166 is predicted to exhibit good intestinal absorption, moderate lipophilicity (AlogP98 = 3.35), and high plasma protein binding, properties which together suggest a favorable passive membrane permeability and a potential for intracellular accumulation beyond what is reflected in the cell-free biochemical assay conditions. And so, at elevated intracellular concentrations, even partial BTK inhibition could be translated into a meaningful downstream pathway suppression in a cellular context. In contrast, compound 168 shows poor intestinal absorption (Level 3) and a substantially higher polar surface area (PSA = 154.5 Å2), which is consistent with its comparatively weaker cellular potency. Moreover, both compounds are predicted to be BBB non-penetrant (Level 4) and non-inhibitors of CYP2D6. Second, the acridine scaffold is a well-established DNA-intercalating pharmacophore [47]. Thus, the acridine derivatives intercalate into double stranded DNA via the planar aromatic stacking mode, and inhibit topoisomerase II activity, which can induce double-strand breaks that independently trigger cell death in rapidly proliferating malignant B-cells such as Raji and K562 lines. Therefore, this mechanism is independent of BTK and may account for a substantial proportion of the observed sub-micromolar antiproliferative activity of compound 166. Third, the off-target kinase activity cannot be excluded, and the BTK ATP-binding pocket shares structural features with other kinases in the B-cell receptor (BCR) signaling cascade, including HCK, SRC-family kinases, and PI3K pathway components [7, 32, 48]. Thus, the anti-BTK hit compounds may engage these targets at cellular concentrations sufficient to produce antiproliferative effects through another supporting signaling pathways, such as PI3K/AKT activation, NF-κB signaling, or PLCG2-mediated calcium mobilization, all of which promote survival and proliferation of malignant B-cells independently of direct BTK catalytic activity. Nonetheless, the measurable BTK enzyme inhibition demonstrated by compounds 166 and 168 supports the utility of our integrated computational and experimental strategy as a starting point for identifying novel anti-BTK candidates.

### Molecular docking analysis of active hits

#### Predicted binding orientation of active hits within the BTK active site

**Fig. 9:**
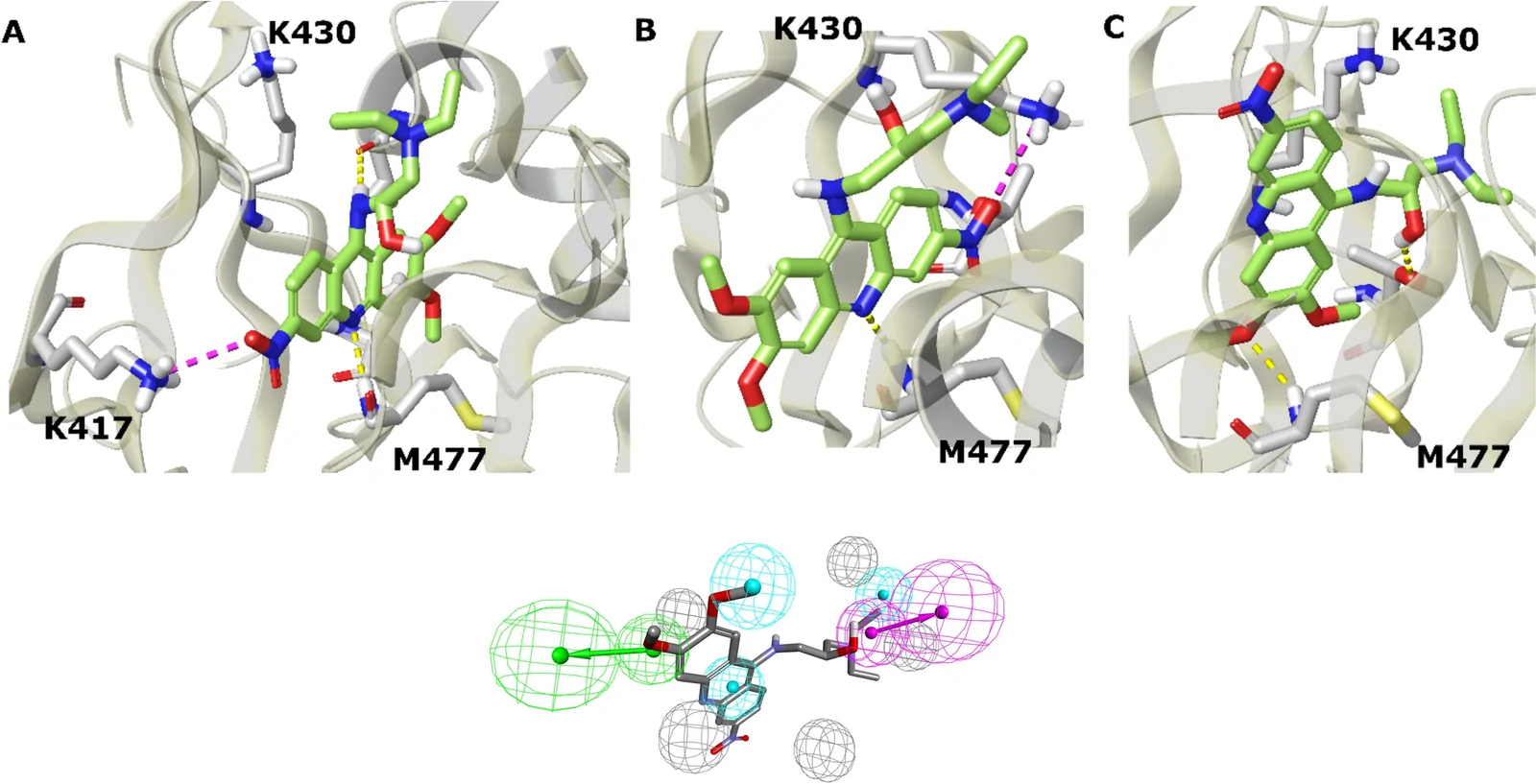
Selected docking poses resulting from Induced Fit Docking. **A** and **B** Hit compound 166 poses its acridine ring horizontal to the hinge region domain while forming H-bond interaction with M477 via its pyridine’s nitrogen. Nitro group faces the solvent accessible binding site in A but facing the catalytic domain in (**B**). **C** Hit compound 166 poses its acridine ring vertical to the hinge region domain while forming H bond interaction with M477 via methoxy group. Thick tube representations for both HIT COMPOUND 166 and selected binding site residues. 166 (Green), BTK (White). Ionic interactions (Magenta dotted lines), H-bond interactions (Yellow dotted lines). **D** Compound 166 mapped against the spatial orientation of functional groups within the pharmacophore GFA-HypoA-T8-10 (Green spheres represent HBA features, blue spheres indicate hydrophobic features, magenta spheres correspond to HBD features, and grey spheres denote exclusion volume regions

Structural analyses of available inhibitor-bound crystal structures of BTK reveals multiple binding modes for the different BTK inhibitors within the ATP-binding site in which all share a common hydrogen bond interaction with hinge region residue (M477). These different binding modes have been reported to influence the conformational state of full length BTK and therefore affecting its activity in B-cell receptor signaling. Based on these structural insights and upon structural comparison of compound 166 with co-crystallized inhibitors, we hypothesized that compound 166 would preferentially adopt a hinge-region only binding mode as resembled in the crystal structures of BTK in complex with BMS-986142 (PDB ID: 5T18) and in complex with BMS-935177 (PDB ID: 5JRS) [15]. To this end, an initial investigation of the potential binding poses of compound 166 inside the BTK active site was performed using InducedFit docking protocol in Schrödinger using three different crystal structures each of which resemble a different binding mode of their co-crystallized inhibitors [44]. (1) PDB ID 6X3N in which the co-crystallized inhibitor sets below the catalytic lysine (K430) and co-occupies the hydrophobic back pocket of the enzyme (including M449, I472, and L542). (2) PDB ID 5VFI in which the co-crystallized ligand sets above the catalytic lysine (K430) and co-occupies the H3 pocket (Q412, F413, L542, S543 and Y551), and 3) PDB ID 5T18 in which the co-crystalized ligand does not engage in hydrogen bond interaction with the catalytic lysine while only occupying the hinge region of the enzyme. Prior to docking, compound 166 was prepared using LigPrep module in Schrödinger, maintaining a neutral state of the tertiary amine group of the compound. This constraint was applied based on available structural information from BTK inhibitor complexes that reveal a solvent accessible binding mode of positively charged moieties, thus minimizing the risk of introducing artificial electrostatic interactions that could bias docking poses as been observed in our prior docking results. OPLS4 force-field dihedral parameters for compound 166 were then evaluated and parameterized using the Force Field builder tool in Schrödinger 2025-2 [45]. Then Induced Fit protocol was performed allowing conformational sampling of compound 166 and prime refinement of protein residues within 5 Å of ligand binding poses. All resulting docking poses were then compared and analyzed visually based on the ability of compound 166 to form the essential hydrogen bond interaction with the hinge region M477. Three of the resulting docking poses were then selected for further evaluation using all atom molecular dynamics simulations where each pose was run in duplicates for 100 ns (Fig. 9).

**Fig. 10:**
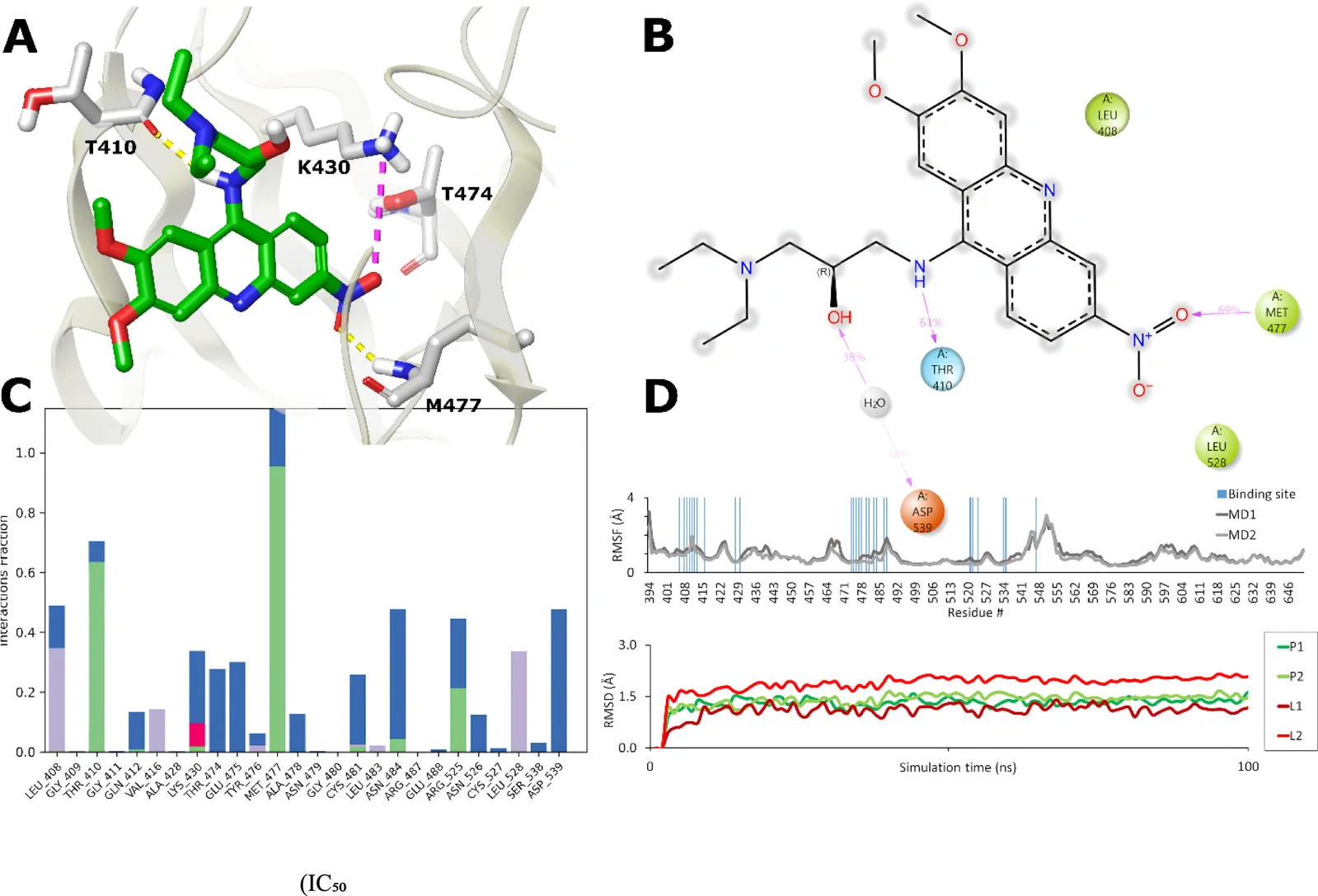
**A** Compound 166 (IC_50_ = 12.2 µM against radiometric HotSpot™ BTK assay) pose within the active site of BTK stabilized during the simulation and showing stable interactions of nitro group at the acridine ring with M477. **B** 2D ligand–protein interaction diagram retrieved from simulation run (MD1). **C** Protein ligand interaction count showing hydrogen bond (green), hydrophobic interactions (Mauve), Water bridging (Blue) and ionic (Magenta) based on simulation run (MD1). **D** Protein RMSF and RMSD retrieved from two simulations based on docking pose 2. (P: Protein, L: Ligand)

The redocking of the co-crystallized ligand (BMS-986142) into the BTK ATP-binding site (PDB ID: 5T18) by using the IFD protocol reproduced the crystallographic binding pose with an RMSD of 0.83 Å (Fig. S1), confirming the accuracy of the docking methodology. IFD of compound 166 into the three BTK crystal structures yielded multiple binding poses. Visual analysis identified three poses that maintained the essential hydrogen bond interaction with the hinge region residue M477 (Fig. 10). In poses A and B, compound 166 oriented its acridine ring horizontally relative to the hinge region domain, forming a hydrogen bond with M477 via its pyridine nitrogen; these two poses differed in the orientation of the nitro group, which faced the solvent-accessible region in pose A and the catalytic domain in pose B. In pose C, the acridine ring was oriented vertically relative to the hinge region, with the hydrogen bond to M477 mediated through the methoxy group.

#### Molecular dynamic results and protein–ligand interactions of hit compound 166 (NCS13003)

The three selected docking poses were each subjected to duplicate 100 ns all-atom MD simulations (six independent simulations total). Analysis of the MD trajectories revealed substantially higher stability of compound 166 in the BTK active site for poses A and B, whereas pose C exhibited rapid ligand migration from the binding site during the simulation. Quantitative hydrogen bond analysis of the simulations based on poses A and B demonstrated a markedly higher propensity for hydrogen bond interactions with M477 in simulations initiated from pose B (occupancy: 94% ± 0.02) compared to those from pose A (occupancy: 3% ± 0.03). Notably, the stable hydrogen bond interaction with M477 in pose B was maintained through the nitro group of the acridine ring rather than through the pyridine nitrogen as observed in the initial docking pose, which indicates a ligand reorientation during the simulation that converged on a more thermodynamically favorable binding mode (Fig. 10A and B).

Further analysis of the protein–ligand interaction profile from the MD trajectories revealed that compound 166 does not form any interactions with the gatekeeper residue T474 (Fig. 10C). This observation provides a structural rationale for the ability of this compound to inhibit the T474I mutant, as the binding mode does not depend on contacts with the gatekeeper residue that would be disrupted by the isoleucine substitution.

Simulations based on docking pose B showed stable protein and ligand RMSD values throughout the 100 ns trajectory, along with consistent RMSF profiles for binding site residues across the duplicate runs (Fig. 10D), supporting the reliability of the predicted binding mode.

### Novelty study on captured hits by applying principal component analysis (PCA) and library comparison analysis

A Principal Component Analysis (PCA) in-silico study by using the DS 4.5 was performed to assess the chemical diversity of the newly captured BTK inhibitors when compared to the dataset used. So, the PCA experiment was performed by using a set of relevant molecular descriptors which included ALogP, Number of Hydrogen Bond Donors, Number of Hydrogen Bond Acceptors, Number of Aromatic Rings, Molecular Surface Area, Molecular Polar Surface Area, and Molecular Fractional Polar Surface Area.

Consequently, the resulting 3D PCA scatter plot (Fig. 8) can visually represent the chemical space that is occupied by our BTK inhibitor dataset and each point in the plot corresponds to a molecule which is positioned according to its principal component scores and the axes. Moreover, PC1, PC2, and PC3, represent the primary dimensions of chemical diversity within the dataset that captures the directions of maximum variance in molecular descriptor space.

Our analysis reveals that PC1 primarily captures diversity related to molecular polarity and hydrogen bonding character, predominantly driven by the Molecular Fractional Polar Surface Area and therefore molecules that show higher PC1 scores tend to exhibit higher fractional polarity and stronger hydrogen bonding potential. Meanwhile, PC2 vector, reflects diversity in aromaticity and, to a lesser extent, hydrophobicity which is largely determined by the number of aromatic rings. And so, higher PC2 scores indicate molecules with greater aromatic content and increased lipophilicity. Finally, PC3 represents a more complex dimension of diversity that contrasts hydrophobicity and hydrogen bond accepting ability against hydrogen bond donating ability and fractional polarity. In conclusion, this PCA novelty study provides a representative visualization of the chemical space spanned by our novel BTK inhibitor hits.

Also, to provide a more direct and quantitative test of the structural novelty of our hit compounds, the “Compare Libraries” protocol in DS 4.5 was used to compare the 165 training and test compounds against the captured active hit compounds across three independent methods, with results summarized as follows: the Bemis–Murcko scaffold (Assemblies) analysis identified 102 total scaffolds across both libraries, of which zero scaffolds were shared between the training/test compounds and the active hits (77 scaffolds unique to the training/test library; 25 scaffolds unique to the active-hit library), which yielded a scaffold-level similarity score of 0.000. This result indicates that none of the captured active hits share a Murcko scaffold with any compound used in model development. Also, the Bayesian model-based comparison yielded a distance of 118.36 between the two libraries, with non-overlapping score distributions: training/test compounds scored positively (average score ≈ 4.52) when evaluated by the Bayesian model trained on their own library but strongly negatively (average score ≈ − 30.31) when evaluated by the model trained on the active-hit library, and vice versa for the active hits (average score ≈ 31.29 under their own model versus ≈ − 52.24 under the training/test model), this confirms that the two libraries occupy distinct regions of Bayesian descriptor space. Finally, the global molecular fingerprint comparison (FCFC_6) showed minimal overlap, with only 174 of 2245 total fingerprint bits shared between the two libraries (1428 bits unique to the training/test library; 643 bits unique to the active-hit library), which correspods to a global fingerprint similarity score of 0.0775. Taken together, these three methodologically independent metrics (scaffold-level, descriptor-space-level, and fingerprint-level) provide quantitative support, beyond the PCA visualization alone, for the structural distinctiveness of the captured active hits relative to the compounds used in model development.

## Discussion

This study demonstrates the effectiveness of the integrated computational and experimental strategy for the discovery of novel Bruton's tyrosine kinase (BTK) inhibitors (see Fig. 2). And so, by combining the QSAR-GFA modeling (Branch 1) with the machine learning classification method (Branch 2), this workflow enabled an efficient prioritization of compounds from the NCI database based on complementary pharmacophoric, physicochemical, and spatial descriptors records (Fig. 2). Accordingly, this convergence between the two independent prediction branches resulted in the successful identification of multiple active hits, in BTK-relevant cell lines, as demonstrated by cytotoxicity assays in Raji lymphoma and K562 leukemia cell lines (Table 7). It should be noted that cytotoxicity in these cell lines is consistent with, but does not exclusively confirm, BTK inhibition as the primary mechanism of action. Raji (Burkitt lymphoma) and K562 (CML) cells express constitutively active BCR signaling components and are therefore relevant models for evaluating compounds that target the BTK pathway; however, they also harbor multiple other oncogenic drivers, including c-Myc translocations (Raji) and BCR-ABL1 (K562), that could independently contribute to antiproliferative responses. Furthermore, as described in Section “Results Correlation between predicted and experimental activity”, the approximately 140-fold discrepancy between the cellular IC_50_ of compound 166 (0.087 µM in Raji cells) and its BTK enzyme IC_50_ (12.2 µM) indicates that alternative mechanisms including acridine-mediated DNA intercalation and off-target kinase activity likely contribute to the observed cellular activity (see Section “Results Correlation between predicted and experimental activity” for full discussion). Further mechanistic studies are required to fully characterize the target engagement of the identified hits.

Therefore, a key outcome of this study is the identification of new chemically distinct, Acridine based, non-covalent BTK inhibitor scaffolds that are different from the previously approved covalent inhibitors (Fig. 1A). And, in contrast to previous BTK inhibitors, which rely on electrophilic sidechains to form irreversible bond with Cys481 in the hinge region, our most potent hit, compound 166 (NCS13003), represents a non-covalent acridine derived chemotype (Fig. 9). The docking analysis results (Section “Molecular Docking Analysis of Active Hits”) show that compound 166 occupies the ATP binding pocket of the BTK enzyme and extends toward the hydrophobic back pocket without adopting a geometry compatible with covalent Cys481 engagement (Fig. 9A–D) and this binding mode enables extensive hydrophobic, π–π, π–alkyl, and electrostatic interactions beyond the classical hinge region which illustrates an alternative strategy for effective BTK inhibition. Also, other active hits, including compounds 167, 168, and 171, similarly exhibit non-covalent binding modes which underscores the structural diversity that has been captured by the integrated screening approach.

Upon analyzing the molecular docking results that are presented in Figs. 9 and 10 (Section “Molecular Docking Analysis of Active Hits”) this provides structural insight into the binding mode of compound 166 within the BTK ATP-binding pocket. Thus, the docking pose reveals hydrogen bonding interactions with Thr474 and Asp539, accompanied with hydrophobic and aromatic interactions with Phe540, Leu460, Ile472, and Met449, and so, these interactions are stabilizing the ligand within the active site which is consistent with the experimentally observed BTK inhibitory activity of compound 166 (IC_50_ = 12.2 µM).

Furthermore, upon comparing the proposed binding mode in Fig. 10 with the pharmacophore mapping scheme in Fig. 9D, we can see a strong spatial alignment between compound 166 and the pharmacophore model GFA-HypoA-T8-10 (ROC-AUC = 0.932). Hence, the planar acridine core occupies the central hydrophobic pharmacophore feature, while the methoxy substituents and flexible side chain that correspond to peripheral hydrogen bond donor/acceptor features. All these agreement points indicate that the pharmacophore model GFA-HypoA-T8-10 (ROC-AUC = 0.932) can successfully capture the key 3D interactions that are required for BTK inhibition.

Moreover, compound 166 interacts with the BTK binding pocket through multiple non-covalent interactions and does not rely on covalent bond formation with Cys481, the residue targeted by all approved irreversible BTK inhibitors (ibrutinib, acalabrutinib, zanubrutinib) [7], the most prevalent resistance mutations in CLL patients arise precisely at C481 (C481S, C481T, C481F, C481R, C481Y, C481G), all of which abrogate covalent inhibitor binding. Additionally, the non-covalent binding mode of compound 166 means that these resistance mutations would not be expected to impair its binding, and the absence of interaction with the gatekeeper residue T474 which is confirmed by the MD simulation data (Section “Molecular Docking Analysis of Active Hits”). It is also suggested that the T474I/F/L gatekeeper mutations, which confer resistance to non-covalent inhibitors such as nemtabrutinib and fenebrutinib, may also not affect compound 166. So, these structural features make compound 166 a promising scaffold for further optimization as a next-generation non-covalent BTK inhibitor candidate, warranting kinase selectivity profiling and direct target engagement validation.

On the other side, the performance of the models developed in this study can be contextualized against another already published BTK prediction models. Previous 3D-QSAR studies have typically been built on congeneric series of BTK inhibitors, including thienopyridine derivatives (CoMFA q2 = 0.574, r^2^ = 0.924; [49]), 2,5-diaminopyrimidines (CoMFA q^2^ = 0.675, r^2^ = 0.961; [49], carbazole carboxamides (CoMFA q^2^ = 0.761, r^2^ = 0.933; 132 compounds; [50]), and pyrrolopyrimidines (CoMFA q^2^ = 0.519, r^2^ = 0.971; 41 compounds; [51]). As seen, these congeneric models achieve high training set correlations (r^2^ = 0.92–0.99) because structural variation is restricted to substitutions within a single scaffold. The GFA-MLR model developed in this study (r^2^ = 0.60, Q^2^ = 0.55) was constructed from a structurally diverse dataset of 165 compounds spanning multiple chemotypes, and the observed fit is consistent with the expected reduction in correlation when modeling across heterogeneous scaffolds. For the classification branch, Li et al. [52] developed ML models for 3895 non-covalent BTK inhibitors and reported a best test accuracy of 94.1% (XGBoost, MCC = 0.75), which is comparable to the Random Forest external accuracy of 94.74% obtained in the present study [53]. Direct quantitative comparison across studies is limited by differences in dataset composition, descriptor types, activity endpoints, and validation protocols. Nonetheless, it is notable that none of the previously published BTK prediction studies advanced their computational hits to prospective experimental validation, which represents a distinguishing feature of this dual-branch workflow.

However, several limitations of the machine learning approach employed in this study should be acknowledged. First, the dataset of 165 BTK inhibitors, while sufficient for preliminary classification modeling, is relatively small for training multiple machine learning algorithms. Also, the learning curve analysis (Supporting Information, Figure E) indicates convergence of cross-validation performance at approximately 45 training compounds (~ 35% of the training set), which provides evidence of dataset adequacy for the Random Forest classifier; however, model performance on structurally distant chemotypes cannot be guaranteed. Second, the use of pharmacophore fit values as ML descriptors introduces a potential circularity, because these features are derived from pharmacophore models that were themselves generated from the same 165-compound set. Although the pharmacophore models and ML classifiers were developed independently within separate workflow branches, the shared compound origin means that the descriptor space is not fully independent of the training data. Third, the high cross-validation performance (κ = 0.99 for Random Forest) on a small dataset carries an inherent risk of overfitting, and the external validation set of 38 compounds, while providing an independent performance estimate (κ = 0.92), has limited statistical power to detect overfitting with high confidence. Fourth, the binary activity classification scheme (active vs. non-active) provides less nuanced information than continuous pIC_50_ regression, and the activity threshold used for classification may influence model performance. Fifth, the SHAP feature importance values reported in this study quantify the statistical contribution of each descriptor to the model’s classification output; the biological interpretations presented in the Discussion represent plausible structure–activity hypotheses that are consistent with, but not mechanistically established by, the SHAP analysis. Finally, this study was restricted to conventional machine learning algorithms (Random Forest, XGBoost, k-NN, Naive Bayes, RProp MLP, and SVM) implemented in KNIME 5.4.0. Deep learning architectures, such as graph neural networks and message-passing neural networks, were not evaluated owing to the limited dataset size, which is generally insufficient to train deep learning models without substantial risk of overfitting. Future studies incorporating larger and more diverse BTK inhibitor datasets may benefit from the application of deep learning methods alongside the conventional approaches used here.

## Conclusions

This study targeted the discovery of novel Bruton's tyrosine kinase (BTK) inhibitors through a dual-branch computational and experimental framework. Additionally, the framework of this study was constructed and validated by using a curated dataset of 165 structurally diverse BTK inhibitors, this was followed by virtual screening of more than 255,000 compounds from the NCI diversity set. Then, by combining the drug design computational insights with the predictive power, the proposed strategy in this study was able to address some key limitations that are related to traditional modelling approaches and thus enabled an efficient exploration of the chemically diverse space beyond that of clinically established BTK inhibitors.

Thus, the efficacy of the convergence of the QSAR-GFA and machine learning branches as drug design approach was proved by the reliable hit prioritization results. As such, the QSAR-GFA model showed good statistical performance (r^2^ = 0.60, Q^2^ = 0.55, r^2^-pred = 0.51) and provided a transparent structure activity relationships in addition to identifying critical physicochemical and pharmacophoric determinants that are related to the BTK inhibition. In parallel, the ensemble machine learning methods, most notably the Random Forest and XGBoost models, achieved excellent predictive performance with cross-validated accuracies values up to 99.3% and Cohen’s κ values up to 0.99 alongside with maintaining strong external validation (κ = 0.92 for Random Forest). Also, these models consistently highlighted some complementary descriptors that are related to dipole orientation, molecular volume, hydrogen bond acidity, and spatial pharmacophoric features. Furthermore, SHAP analysis was used to identify the most statistically influential descriptors for the model’s classification output. Therefore, when the descriptors are interpreted in the context of known BTK structural biology, a coherent pharmacological picture emerges. For example, the dipole moment along the Y-axis (Dipole-Y-DMol3) descriptor which emerged as the highest-ranked feature, is consistent with the electrostatic complementarity that is required for the effective binding inside the BTK kinase domain. The crystallographic studies of both covalent and non-covalent BTK inhibitors demonstrate that the ATP binding pocket is defined by the directional hydrogen bonds to the hinge region residues (E475 and M477) [7], and a correctly oriented molecular dipole would favor the electrostatic alignment necessary for these interactions. Additionally, the high importance of pharmacophoric fit values (HypoB-T2-5 and HypoH-T8-4) further reinforces this interpretation and all in all these pharmacophores encode the 3D arrangement of hydrogen bond donor/acceptor and hydrophobic features that collectively represent the spatial constraints of the BTK binding site. Notably, HypoB-T2-5 achieved perfect discrimination in the extended ROC validation (AUC = 1.000, BEDROC = 1.000; Table 4), which provides independent evidence that the spatial pharmacophoric pattern captured by this pharmacophore reflects a genuine BTK-active structural requirements rather than just being a statistical artifact.

Also, the molecular surface area and the molecular volume descriptors, which exhibited substantial SHAP contributions, are interpretable in the context of BTK binding site topology. And so, the ATP binding pocket of BTK accommodates inhibitors that are sufficiently large to span from the hinge region to the selectivity pocket yet compact enough to avoid steric clashes with the gatekeeper residue T474 and the surrounding DFG motif [7]. Therefore, inhibitors with excessive molecular volume or surface area may encounter steric occlusion, consistent with the negative SHAP values observed for outlier descriptor values in the summary plot (Fig. 7). The moderate but consistent importance of hydrogen bond acidity aligns with the established role of hydrogen bond donor functionalities in engaging the backbone carbonyl of M477 in the hinge region and the catalytic lysine K430, both of which are critical contact points in the binding of clinically advanced non-covalent BTK inhibitors such as pirtobrutinib and fenebrutinib [7]. Collectively, the convergence of electrostatic (dipole orientation), steric (molecular surface area and volume), and pharmacophoric (hydrogen bond acidity and three-dimensional fit) descriptors in the SHAP hierarchy mirrors the multi-point recognition pharmacology of the BTK ATP-binding pocket and lends pharmacological coherence to the Random Forest model’s predictive framework.

It is noteworthy that the SHAP identified descriptors are predominantly non-covalent interaction features (electrostatic complementarity, hydrogen bonding capacity, and shape) rather than descriptors encoding covalent reactivity. This is consistent with the non-covalent binding mode of the acridine hit compounds which are identified in this study and may have implications for resistance-mutation contexts: because C481 mutations (C481S, C481T, C481F, C481R, C481Y) abrogate the covalent warhead engagement that is required by irreversible inhibitors [7], Furthermore, compounds whose predicted activity depends on the non-covalent descriptor features highlighted by SHAP may retain activity against the C481 mutant BTK. However, it is important to note that SHAP values are able to quantify the predictive importance within the statistical model and that the biological interpretations that are presented here represent a plausible structure activity hypotheses that are consistent with, the SHAP analysis, yet they require experimental validation through structure activity relationship studies and biophysical binding assays.Furthermore, the application of our proposed framework to virtual screening against the NCI database resulted in the prioritization of 28 candidate compounds to be experimentally validated. As a result, cytotoxicity assays in Raji lymphoma and K562 leukemia cell lines revealed antiproliferative activity spanning the sub-micromolar to low-micromolar range, with IC_50_ values extending from approximately 0.087–22 µM. Remarkably, the most potent compound, 166 (NCS13003), exhibited a nanomolar activity in Raji cells (IC_50_ = 0.087 µM) and a sub-micromolar activity in K562 cells (IC_50_ = 0.73 µM). Moreover, compound, 166 (NCS13003) is representing a chemically distinct, non-covalent heteroaromatic based BTK inhibitor that interacts with the ATP binding pocket through a network of hydrogen bonding, π–π stacking, and hydrophobic interactions rather than covalent modification of Cys481.

Consequently, identifying non-covalent BTK inhibitor scaffolds is particularly significant in the context of clinically relevant resistance mechanisms associated with Cys481 mutations, which compromise the efficacy of irreversible inhibitors. The docking analysis in this study suggests that none of the validated hits relied on covalent cysteine engagement. Additionally, the identification of compound (171) which accommodate a nitrogen mustard containing scaffold with low-micromolar cytotoxic activity (IC_50_ = 11.23 µM on Raji cells) highlights the capacity of our proposed workflow to reveal an unconventional chemotypes with potential BTK associated activity beyond their classical DNA alkylating roles.

Overall, our presented dual-branch strategy which is combining QSAR-GFA, machine learning, pharmacophore modeling, virtual screening, molecular docking, and cytotoxicity assays provides a robust workflow for kinase-targeted drug discovery. The identified acridine-based compounds, most notably compound 166, represent promising antiproliferative candidates with measurable BTK inhibitory activity that still need further investigation. Future studies should include target engagement assays, kinase selectivity profiling, and mechanistic studies to clarify the primary mechanism of action and to determine the extent to which BTK inhibition contributes to the observed cellular effects.

## Supplementary Information

Below is the link to the electronic supplementary material.Supplementary file1 (DOCX 3754 kb)

## Data Availability

No datasets were generated or analysed during the current study.
