# Supplementary material for: Discovery of novel acridine based inhibitors of Bruton’s tyrosine kinase (BTK) via pharmacophore modeling and machine learning-driven virtual screening followed by molecular dynamic studies
Source: J Comput Aided Mol Des. 2026 Sep 1;40(1):222. doi: 10.1007/s10822-026-00912-4 (PMC13534166; doi:10.1007/s10822-026-00912-4)
Supplement: Supplementary file 1 [file 10822_2026_912_MOESM1_ESM.docx]

**Supporting Information**

**Discovery of Novel Acridine based Inhibitors of Bruton’s Tyrosine Kinase (BTK) via Pharmacophore Modeling and Machine Learning-Driven Virtual Screening followed by Molecular Dynamic Studies**

*Rand Shahin*, *^[a]^ *Sawsan Jaafreh*, ^[b]^*,* Rufaida Al Zoubi, ^[c]^ *Iman Mansi*, ^[d]^ *Bashaer Abu-Irmaileh,* ^[e]^ *Yusra Azzam,* ^[f]^ *Salma Azzam*, ^[f]^

[a]	Department of Pharmaceutical Chemistry, Faculty of Pharmaceutical sciences, The Hashemite University, P.O. Box 330117, Zarqa, 13133 Jordan. ORCID: https://orcid.org/0000-0001-7330-4433Phone: 00962 799-799-044. e-mail: r.shahin@hu.edu.jo

[b]	Department of Chemistry, Faculty of Science, The Hashemite University. P.O. Box 330117, Zarqa, 13133 Jordan.

[c]	Department of Medicinal Chemistry and Pharmacognosy, Faculty of Pharmacy, Jordan University of Science & Technology, Irbid, Jordan

[d]	Department of Clinical Pharmacy and Pharmacy Practice, Faculty of Pharmaceutical Sciences, The Hashemite University, Zarqa, Jordan.

[d]	Hamdi Mango Center, The University of Jordan, Amman, Jordan.

[e]	Jacobs School of Medicine and Biomedical Sciences, University at Buffalo, 955 Main Street, Buffalo NY 14203.

**Supporting material document content**

| **Pages 2-5** | **S1, S2 and S3** |
| --- | --- |
| **Pages 6- 35** | **Table A** |
| **Page 36** | **Table B** |
| **Page 37-38** | **Table C** |
| **Page 39** | **Figure A** |
| **Page 40-42** | **Figure B** |
| **Page 43** | **Figure C** |
| **Page 44** | **Figure D** |
| **Page 45** | **Figure S1** |

**Section S1: Pharmacophore Modeling for Bruton’s Tyrosine Kinase (BTK) by Using the CATALYST Algorithm**

In this study, CATALYST algorithm was utilized to generate the supervised pharmacophoric models specific to Bruton’s Tyrosine Kinase (BTK) and to find new hit compounds to treat B-cell malignancies. Subsequently, during the pharmacophore generation step the modeling process involved three systematic phases: the constructive, the subtractive, and the optimization steps, also all phases were coupled with a comprehensive cost analysis to identify robust pharmacophore hypotheses.

**1. The Constructive Phase**

This phase involved inspecting the training dataset and aligning the most active BTK inhibitors based on the most prominent shared chemical features. As for the modeling parameters, the no. of features was allowed to be up to five. And the activity of BTK inhibitors was evaluated by using Equation A:

(Most Active × Uncertainty of Most Active) – (CompAct / Unc) > 0.0

Where:

- Most Active: Activity of the most potent BTK inhibitor in the training set.
- Uncertainty of Most Active: Uncertainty associated with Most Active.
- CompAct: Activity of the compound under evaluation.
- Unc: Uncertainty of the evaluated compound.

Moreover, if more than eight compounds met the criteria for high activity, only the top eight were used for alignment. Accordingly, these alignments formed the basis of pharmacophore hypothesis generation.

**2. The Subtractive Phase**

Next, the algorithm removed the hypotheses that were explaining inactivity in BTK inhibitors that is to ensure specificity. A BTK inhibitor was classified as inactive if it satisfied Equation B:

Log (CompAct)−log (Most Activ)>3.0

The subtractive step can reduce the likelihood of false positives and make the resulted hypotheses more selective towards active BTK binding modes.

**3. The Optimization Phase**

The generated hypotheses were refined to enhance their predictive accuracy and to correlate better with the BTK inhibitor bioactivity. These modifications includes the following:

- Rotating the pharmacophoric features.
- Adding or removing features aiming towards better alignment.

This iterative refinement process during the optimization step also aims to maximize 3D quantitative structure activity relationship (3D-QSAR) properties of the hypotheses. Then, the top 10 pharmacophore hypotheses are ranked by their performance metrics, and finally selected as optimal models during the pharmacophore generation run.

**Section S2: Implementing the Subset Generation Technique during the BTK Inhibitor Study as Preliminary Step for Pharmacophore Generation.**

A diversity driven subset generation step from the major training set in table A under supplemental material was employed to initiate the process of pharmacophore modeling for BTK inhibitors. Subsequently, the "active group" of BTK inhibitors in Table A (compounds 1-165) were identified based on their inhibitory activities (IC_50_ values for active compounds should range from 1.0 to 9.0 nM in this study).

Then, a diversity analysis was conducted on the selected "active group" by using the "Find Diverse Molecules" protocol within Discovery Studio 4.5 (DS 4.5). And, the aim of this step is to select a set of diverse representatives, or "seeds," from this active group to guide the subsequent partitioning of the full dataset. The molecular properties that were employed in this diversity analysis included FCFP_6 fingerprints, hydrogen bond donor and acceptor count, ring counts, polar and accessible surface areas, as well as fragment and atom count. Thus, this diverse property set aimed to capture a broad spectrum of structural and physicochemical characteristics relevant to BTK inhibitors.

As a result, 8 diverse, highly active compounds were selected as "seeds." (see table B and Figure A in the Supplemental material). These "seed" compounds then played a crucial role in partitioning the entire dataset of BTK inhibitors into more homogeneous subsets (Figure A under supplementary material). This partitioning was achieved through a sequential mapping process that is guided by the screening maps derived from these diverse active BTK inhibitors (Figure A). Consequently, the sequential mapping comprised several stages: (1) selecting the highly active compounds in the subset that are fully matching the seed-derived maps; then (2) selecting the intermediately active compounds in the subset that are exhibiting a match with one missing feature; alongside (3) selecting, the non-active compounds that failed to map to the seed derived maps in figure A; and finally (4) eliminating the outlier compounds that were identified by their significant activity deviations during subsequent QSAR model development. Finally, each of these refined subsets in table B under supplemental material were generated through this diversity driven partitioning and was then utilized for supervised pharmacophore generation. This was accomplished using the Catalyst HypoGen module within Discovery Studio 4.5 by employing the "3D QSAR Pharmacophore Generation" protocol. Finally, specific parameters for the pharmacophore generation step included the "Best" conformation generation option, a minimum inter-feature distance of 2.97 Å, and a fixed inactivity spread of about three orders of magnitude.

**Section S3: 3D QSAR Pharmacophore Generation and Validation Steps:**

The “3D QSAR pharmacophore generation” protocol was implemented in DS 4.5 through sixty-four modeling runs (Table C, supplemental material). This protocol is tailored to design pharmacophores that are predictive and validated from the eight sets of previously known ligands (as shown in table B under supplemental material).

The pharmacophore generation algorithm operates through three sequential phases: the constructive phase, the subtractive phase, and the optimization phase which ensures that the pharmacophoric models are characteristic of the active ligands but not the inactive ones. This exploration method, which has been extensively validated in the literature [29–35], was further refined by adjusting the inter-feature spacing parameter between 100 and 300 pm. Additionally, the maximum number of features in the resulting hypotheses was flexibly varied, by alternating between 4 to 5 or 5 to 5 features.

The “3D QSAR pharmacophore generation” protocol integrates HypoGen to derive structure-activity relationship (SAR) insights from datasets where activity values (e.g., IC_50_) span at three orders of magnitude, which offers a robust basis for model generation. The “3D QSAR pharmacophore generation in this study also supports the inclusion of excluded volume features derived from inactive compounds to improve the accuracy.

Furthermore, the statistical validation using Fisher analysis and confidence levels (90%) were employed to enhance the reliability of the results. Also, parallel processing options were used to enable distributed computation for large datasets

These validation and computational efforts aimed to investigate the underlying pharmacophoric features that are contributing to BTK kinase activity which provided critical insights into novel anticancer drug design and discovery.

- **Cost Analysis**

The quality of generated pharmacophore hypotheses was assessed by using a cost evaluation system comprising three components (Weight cost, Error cost, and Configuration cost):

- **Weight Cost**

This metric penalizes deviations in feature weights from the ideal value of 2 which ensures balanced contributions from the various chemical features within the hypothesis.

- **Error Cost**

Error cost quantifies the discrepancy between experimental and estimated activities of BTK inhibitors. Estimated activity was calculated by using the Equation :

Log (Estimated Activity) =I +Fit log

Where I is the regression intercept, and Fit is derived from Equation D:

Fit=∑ (mapped features) × W [1−∑(disp/tol)^2^]

Here:

- disp: Distance between the feature center and corresponding chemical moiety.
- tol: Tolerance radius (default 1.6 Å).
- W: Weight of the feature.

The summation included all pharmacophoric features that successfully aligned with corresponding functionalities of the BTK inhibitors.

- **Configuration Cost**

Configuration cost captures the complexity of the hypotheses, with higher feature counts (up to five) leading to increased entropy and cost.

- **Total Cost and Null Hypothesis**

The total cost, combining weight, error, and configuration costs is next compared to the null hypothesis cost, Thus greater divergence from the null hypothesis cost indicates a statistically significant pharmacophore hypotheses.

- **Model Selection**

The generated hypotheses are to be ranked based on their resulted total cost. Then, the models with lower total costs and significant divergence from the null hypothesis are to be prioritized. Finally, the top 10 pharmacophoric models are to be selected as the most robust representations of BTK-binding pharmacophore features.

| **Table A**: The structures of BTK inhibitors utilized in modeling. | | | | | |
| --- | --- | --- | --- | --- | --- |
|  | | | | | |
| **No.** | **Reference** | **R_1_** | **R_2_** | **X** | **IC_50_ (µM)** |
| 1 | (Caldwell et al., 2018) |  |  | C | 0.011 |
| 2 | (Caldwell et al., 2018) |  |  | C | 0.0015 |
| 3 | (Caldwell et al., 2018) |  |  | C | 1.6 |
| 4 | (Caldwell et al., 2018) |  |  | C | 0.0011 |

| **Table A**: The structures of BTK inhibitors utilized in modeling. | | | | | |
| --- | --- | --- | --- | --- | --- |
|  | | | | | |
| **No.** | **Reference** | **R_1_** | **R_2_** | **X** | **IC_50_ (µM)** |
| 5 | (Caldwell et al., 2018) |  |  | CF | 0.0016 |
| 6 | (Caldwell et al., 2018) |  |  | C | 0.0019 |
| 7 | (Caldwell et al., 2018) |  |  | C | 0.0013 |
| 8 | (Caldwell et al., 2018) |  |  | C | 0.016 |

| **Table A**: The structures of BTK inhibitors utilized in modeling. | | | |
| --- | --- | --- | --- |
|  | | | |
| **No.** | **Reference** | | **IC_50_ (µM)** |
| 9 | (Crawford et al., 2020) | | 0.36 |
|  | | | |
| **No.** | **Reference** | **R** | **IC_50_ (µM)** |
| 10 | (Crawford et al., 2020) |  | 0.0071 |
| 11 | (Crawford et al., 2020) |  | 0.015 |
| 12 | (Crawford et al., 2020) |  | 0.014 |
| 13 | (Crawford et al., 2020) |  | 0.044 |
| 14 | (Crawford et al., 2020) |  | 0.2 |
| 15 | (Crawford et al., 2020) |  | 0.012 |
| 16 | (Crawford et al., 2020) |  | 0.022 |
| 17 | (Crawford et al., 2020) |  | 0.074 |
| 18 | (Crawford et al., 2020) |  | 0.074 |
| 19 | (Crawford et al., 2020) |  | 0.32 |
| 20 | (Crawford et al., 2020) |  | 0.06 |
| 21 | (Crawford et al., 2020) |  | 0.0023 |

| **Table A**: The structures of BTK inhibitors utilized in modeling. | | | |
| --- | --- | --- | --- |
|  | | | |
| **No.** | **Reference** | **R** | **IC_50_ (µM)** |
| 22 | (Crawford et al., 2020) |  | 0.0038 |
|  | | | |
| **No.** | **Reference** | **R** | **IC_50_ (µM)** |
| 23 | (Crawford et al., 2020) |  | 0.098 |
| 24 | (Crawford et al., 2020) |  | 0.0077 |
| 25 | (Crawford et al., 2020) |  | 0.0048 |

| **Table A**: The structures of BTK inhibitors utilized in modeling. | | | | | |
| --- | --- | --- | --- | --- | --- |
|  | | | | | |
| **No.** | **Reference** | **R** | **X** | **Y** | **IC_50_ (µM)** |
| 26 | (Crawford et al., 2020) |  | CH | CH | 0.0032 |
| 27 | (Crawford et al., 2020) |  | CH | CH | 0.0024 |
| 28 | (Crawford et al., 2020) |  | CH | CH | 0.032 |
| 29 | (Crawford et al., 2020) |  | CH | CH | 0.0072 |
| 30 | (Crawford et al., 2020) |  | N | CH | 0.026 |
| 31 | (Crawford et al., 2020) |  | CH | N | 0.0051 |

| **Table A**: The structures of BTK inhibitors utilized in modeling. | | |
| --- | --- | --- |
|  | | |
| **No.** | **Reference** | **IC_50_ (µM)** |
| 32 | (Crawford et al., 2020) | 0.41 |
|  | | |
| **No.** | **Reference** | **IC_50_ (µM)** |
| 33 | (Crawford et al., 2020) | 0.28 |
|  | | |
| **No.** | **Reference** | **IC_50_ (µM)** |
| 34 | (Crawford et al., 2020) | 0.002 |

| **Table A**: The structures of BTK inhibitors utilized in modeling. | | |
| --- | --- | --- |
|  | | |
| **No.** | **Reference** | **IC_50_ (µM)** |
| 35 | (de Bruin et al., 2020) | 0.018 |
|  | | |
| **No.** | **Reference** | **IC_50_ (µM)** |
| 36 | (de Bruin et al., 2020) | 0.0072 |

| **Table A**: The structures of BTK inhibitors utilized in modeling. | | |
| --- | --- | --- |
|  | | |
| **No.** | **Reference** | **IC_50_ (µM)** |
| 37 | (de Bruin et al., 2020) | 0.0035 |
|  | | |
| **No.** | **Reference** | **IC_50_ (µM)** |
| 38 | (de Bruin et al., 2020) | 0.0012 |

| **Table A**: The structures of BTK inhibitors utilized in modeling. | | | | |
| --- | --- | --- | --- | --- |
|  | | | | |
| **No.** | **Reference** | **R** | | **IC_50_ (µM)** |
| 39 | (de Bruin et al., 2020) |  | | 0.0072 |
| 40 | (de Bruin et al., 2020) |  | | 0.0047 |
| 41 | (de Bruin et al., 2020) |  | | 0.0025 |
|  | | | | |
| **No.** | **Reference** | **R_1_** | **R_2_** | **IC_50_ (µM)** |
| 42 | (de Bruin et al., 2020) |  |  | 0.0015 |
| 43 | (de Bruin et al., 2020) |  |  | 0.0038 |
| 44 | (de Bruin et al., 2020) |  |  | 0.001 |
| 45 | (de Bruin et al., 2020) |  |  | 0.0016 |

| **Table A**: The structures of BTK inhibitors utilized in modeling. | | |
| --- | --- | --- |
|  | | |
| **No.** | **Reference** | **IC_50_ (µM)** |
| 46 | (Kim et al., 2011) | 0.00252 |
|  | | |
| **No.** | **Reference** | **IC_50_ (µM)** |
| 47 | (Kim et al., 2011) | 0.0191 |
|  | | |
| **No.** | **Reference** | **IC_50_ (µM)** |
| 48 | (Kim et al., 2011) | 0.00399 |

| **Table A**: The structures of BTK inhibitors utilized in modeling. | | | | | |
| --- | --- | --- | --- | --- | --- |
|  | | | | | |
| **No.** | **Reference** | **R_1_** | **X** | **R_2_** | **IC_50_ (µM)** |
| 49 | (Kim et al., 2011) | H | Cl | No aryl group | 4.29 |
| 50 | (Kim et al., 2011) | H | N | 2-Me, 5-F | 0.00333 |
| 51 | (Kim et al., 2011) | H | N | 2-Me, 4-F | 0.00296 |
| 52 | (Kim et al., 2011) | H | N | 2-Me, 3-F | 0.00189 |
| 53 | (Kim et al., 2011) | H | N | 2-Me, 6-Cl | 0.0103 |
| 54 | (Kim et al., 2011) | H | N | 2-Me, 5-Cl | 0.0132 |
| 55 | (Kim et al., 2011) | H | N | 2-Me, 4-Cl | 0.00756 |
| 56 | (Kim et al., 2011) | H | N | 2-Me, 3-Cl | 0.0606 |
| 57 | (Kim et al., 2011) | H | N | 2-Me, 6-OH | 0.0104 |
| 58 | (Kim et al., 2011) | H | N | 2-Me, 5-OH | 0.00155 |
| 59 | (Kim et al., 2011) | H | N | 2-Me, 4-OH | 0.0122 |
| 60 | (Kim et al., 2011) | H | N | 2-Me, 3-OH | 0.0765 |
| 61 | (Kim et al., 2011) | F | N | 2-Me | 0.00193 |
| 62 | (Kim et al., 2011) | F | N | 2-Cl | 0.00182 |
| 63 | (Kim et al., 2011) | F | N | 2-Me, 5-F | 0.00676 |
| 64 | (Kim et al., 2011) | F | N | 2-Me, 4-F | 0.00535 |
| 65 | (Kim et al., 2011) | Cl | N | 2-Me | 0.00483 |
| 66 | (Kim et al., 2011) | Cl | N | 2-Cl | 0.00503 |
| 67 | (Kim et al., 2011) | Cl | N | 2-Me, 5-F | 0.00398 |
| 68 | (Kim et al., 2011) | Cl | N | 2-Me, 4-F | 0.00953 |
| 69 | (Kim et al., 2011) | F | O | 2-Me | 0.00551 |
| 70 | (Kim et al., 2011) | F | O | 2-Me, 5-F | 0.013 |

| **Table A**: The structures of BTK inhibitors utilized in modeling. | | | | | |
| --- | --- | --- | --- | --- | --- |
|  | | | | | |
| **No.** | **Reference** | **R_1_** | **X** | **R_2_** | **IC_50_ (µM)** |
| 71 | (Kim et al., 2011) | F | O | 2-Me, 4-F | 0.00953 |
| 72 | (Kim et al., 2011) | H | O | 2-Me, 4-F | 0.00658 |
| 73 | (Kim et al., 2011) | H | S | 2-Me | 0.283 |
|  | | | | | |
| **No.** | **Reference** | **R_1_** | **X** | **R_2_** | **IC_50_ (µM)** |
| 74 | (Kim et al., 2011) | H | N | 2-Me, 5-F | 0.072 |
| 75 | (Kim et al., 2011) | H | O | 2-Me, 4-F | 1.22 |
| 76 | (Kim et al., 2011) | F | N | 2-Me | 0.04 |
| 77 | (Kim et al., 2011) | F | O | 2-Me | 0.67 |

| **Table A**: The structures of BTK inhibitors utilized in modeling. | | | |
| --- | --- | --- | --- |
|  | | | |
| **No.** | **Reference** | **R** | **IC_50_ (µM)** |
| 78 | (Pan et al., 2007) |  | 0.02 |
| 79 | (Pan et al., 2007) |  | 0.0036 |
| 80 | (Pan et al., 2007) |  | 0.0016 |
| 81 | (Pan et al., 2007) |  | 0.0014 |
| 82 | (Pan et al., 2007) |  | 0.0025 |

| **Table A**: The structures of BTK inhibitors utilized in modeling. | | | |
| --- | --- | --- | --- |
|  | | | |
| **No.** | **Reference** | **R** | **IC_50_ (µM)** |
| 83 | (Qiu et al., 2018) |  | 0.0075 |
| 84 | (Qiu et al., 2018) |  | 0.0022 |
| 85 | (Qiu et al., 2018) |  | 0.0023 |
| 86 | (Qiu et al., 2018) |  | 0.0013 |
| 87 | (Qiu et al., 2018) |  | 0.0019 |
| 88 | (Qiu et al., 2018) |  | 0.0013 |

| **Table A**: The structures of BTK inhibitors utilized in modeling. | | |
| --- | --- | --- |
|  | | |
| **No.** | **Reference** | **IC_50_ (µM)** |
| 89 | (Shi et al., 2014) | 0.067 |
|  | | |
| **No.** | **Reference** | **IC_50_ (µM)** |
| 90 | (Shi et al., 2014) | 12.7 |
|  | | |
| **No.** | **Reference** | **IC_50_ (µM)** |
| 91 | (Shi et al., 2014) | 0.15 |

| **Table A**: The structures of BTK inhibitors utilized in modeling. | | |
| --- | --- | --- |
|  | | |
| **No.** | **Reference** | **IC_50_ (µM)** |
| 92 | (Shi et al., 2014) | > 50 |
|  | | |
| **No.** | **Reference** | **IC_50_ (µM)** |
| 93 | (Shi et al., 2014) | > 50 |
|  | | |
| **No.** | **Reference** | **IC_50_ (µM)** |
| 94 | (Shi et al., 2014) | 0.038 |

| **Table A**: The structures of BTK inhibitors utilized in modeling. | | |
| --- | --- | --- |
|  | | |
| **No.** | **Reference** | **IC_50_ (µM)** |
| 95 | (Shi et al., 2014) | 0.213 |
|  | | |
| **No.** | **Reference** | **IC_50_ (µM)** |
| 96 | (Shi et al., 2014) | 0.574 |

| **Table A**: The structures of BTK inhibitors utilized in modeling. | | | |
| --- | --- | --- | --- |
|  | | | |
| **No.** | **Reference** | **R** | **IC_50_ (µM)** |
| 97 | (Shi et al., 2014) |  | 0.064 |
| 98 | (Shi et al., 2014) |  | 0.025 |
| 99 | (Shi et al., 2014) |  | 0.028 |
| 100 | (Shi et al., 2014) |  | 0.017 |
| 101 | (Shi et al., 2014) |  | 0.143 |
| 102 | (Shi et al., 2014) |  | 0.043 |
| 103 | (Shi et al., 2014) |  | 0.087 |
| 104 | (Shi et al., 2014) |  | 0.126 |
| 105 | (Shi et al., 2014) |  | 0.019 |

| **Table A**: The structures of BTK inhibitors utilized in modeling. | | | |
| --- | --- | --- | --- |
|  | | | |
| **No.** | **Reference** | **R** | **IC_50_ (µM)** |
| 106 | (Shi et al., 2014) | H | 0.008 |
| 107 | (Shi et al., 2014) |  | 0.17 |
| 108 | (Shi et al., 2014) |  | 0.14 |
| 109 | (Shi et al., 2014) |  | > 50 |
| 110 | (Shi et al., 2014) |  | 29 |
| 111 | (Shi et al., 2014) |  | 0.97 |

| **Table A**: The structures of BTK inhibitors utilized in modeling. | | |
| --- | --- | --- |
|  | | |
| **No.** | **Reference** | **IC_50_ (µM)** |
| 112 | (Shi et al., 2014) | 0.033 |
|  | | |
| **No.** | **Reference** | **IC_50_ (µM)** |
| 113 | (Shi et al., 2014) | 0.065 |
|  | | |
| **No.** | **Reference** | **IC_50_ (µM)** |
| 114 | (Shi et al., 2014) | 0.026 |

| **Table A**: The structures of BTK inhibitors utilized in modeling. | | | |
| --- | --- | --- | --- |
|  | | | |
| **No.** | **Reference** | **R** | **IC_50_ (µM)** |
| 115 | (Shi et al., 2014) |  | 0.032 |
| 116 | (Shi et al., 2014) |  | 0.048 |
| 117 | (Shi et al., 2014) |  | 0.083 |
| 118 | (Shi et al., 2014) |  | 0.169 |
| 119 | (Shi et al., 2014) |  | 0.109 |

| **Table A**: The structures of BTK inhibitors utilized in modeling. | | | |
| --- | --- | --- | --- |
|  | | | |
| **No.** | **Reference** | **R** | **IC_50_ (µM)** |
| 120 | (Shi et al., 2014) | NH_2_ | 0.077 |
| 121 | (Shi et al., 2014) | Me | 0.035 |
| 122 | (Shi et al., 2014) |  | 0.018 |
| 123 | (Shi et al., 2014) |  | 0.049 |
| 124 | (Shi et al., 2014) |  | 0.027 |
| 125 | (Shi et al., 2014) |  | 0.041 |

| **Table A**: The structures of BTK inhibitors utilized in modeling. | | | |
| --- | --- | --- | --- |
|  | | | |
| **No.** | **Reference** | **R** | **IC_50_ (µM)** |
| 126 | (Smith et al., 2015) | H | 3.5 |
| 127 | (Smith et al., 2015) |  | 0.24 |
| 128 | (Smith et al., 2015) |  | 0.1 |
| 129 | (Smith et al., 2015) |  | 0.85 |
| 130 | (Smith et al., 2015) |  | 0.012 |
| 131 | (Smith et al., 2015) |  | 0.004 |

| **Table A**: The structures of BTK inhibitors utilized in modeling. | | |
| --- | --- | --- |
|  | | |
| **No.** | **Reference** | **IC_50_ (µM)** |
| 132 | (Watterson et al., 2016) | 0.003 |
|  | | |
| **No.** | **Reference** | **IC_50_ (µM)** |
| 133 | (Watterson et al., 2016) | 0.044 |
|  | | |
| **No.** | **Reference** | **IC_50_ (µM)** |
| 134 | (Watterson et al., 2016) | 0.0051 |

| **Table A**: The structures of BTK inhibitors utilized in modeling. | | |
| --- | --- | --- |
|  | | |
| **No.** | **Reference** | **IC_50_ (µM)** |
| 135 | (Watterson et al., 2016) | 0.0026 |
|  | | |
| **No.** | **Reference** | **IC_50_ (µM)** |
| 136 | (Watterson et al., 2016) | 0.0028 |
|  | | |
| **No.** | **Reference** | **IC_50_ (µM)** |
| 137 | (Watterson et al., 2016) | 0.001 |

| **Table A**: The structures of BTK inhibitors utilized in modeling. | | |
| --- | --- | --- |
|  | | |
| **No.** | **Reference** | **IC_50_ (µM)** |
| 138 | (Watterson et al., 2016) | 0.13 |
|  | | |
| **No.** | **Reference** | **IC_50_ (µM)** |
| 139 | (Watterson et al., 2016) | 0.0016 |

| **Table A**: The structures of BTK inhibitors utilized in modeling. | | | | |
| --- | --- | --- | --- | --- |
|  | | | | |
| **No.** | **Reference** | **R_1_** | **R_2_** | **IC_50_ (µM)** |
| 140 | (Watterson et al., 2016) | H |  | 0.011 |
| 141 | (Watterson et al., 2016) | Cl |  | 0.0026 |
| 142 | (Watterson et al., 2016) | F |  | 0.0022 |

| **Table A**: The structures of BTK inhibitors utilized in modeling. | | | |
| --- | --- | --- | --- |
|  | | | |
| **No.** | **Reference** | **R** | **IC_50_ (µM)** |
| 143 | (Hopkins et al., 2019) |  | 11 |
| 144 | (Hopkins et al., 2019) |  | 16 |
| 145 | (Hopkins et al., 2019) |  | 19 |
| 146 | (Hopkins et al., 2019) |  | 12 |
| 147 | (Hopkins et al., 2019) |  | 8.3 |
| 148 | (Hopkins et al., 2019) |  | 2 |
| 149 | (Hopkins et al., 2019) |  | 0.8 |

| **Table A**: The structures of BTK inhibitors utilized in modeling. | | | |
| --- | --- | --- | --- |
|  | | | |
| **No.** | **Reference** | **R** | **IC_50_ (µM)** |
| 150 | (Hopkins et al., 2019) |  | 4.9 |
| 151 | (Hopkins et al., 2019) |  | 19 |
| 152 | (Hopkins et al., 2019) |  | 45 |
| 153 | (Hopkins et al., 2019) |  | 4.1 |
| 154 | (Hopkins et al., 2019) |  | 44 |
| 155 | (Hopkins et al., 2019) |  | 68 |

| **Table A**: The structures of BTK inhibitors utilized in modeling. | | | | | |
| --- | --- | --- | --- | --- | --- |
|  | | | | | |
| **No.** | **Reference** | **R_1_** | **R_2_** | **R_3_** | **IC_50_ (µM)** |
| 156 | (Hopkins et al., 2019) | N | H | H | 2.7 |
| 157 | (Hopkins et al., 2019) | H | N | H | 3.2 |
| 158 | (Hopkins et al., 2019) | H | H | N | > 10 |
| 159 | (Hopkins et al., 2019) | F | H | H | 0.5 |
| 160 | (Hopkins et al., 2019) | H | F | H | 1.2 |
| 161 | (Hopkins et al., 2019) | H | H | F | 1.3 |
| 162 | (Hopkins et al., 2019) | Cl | H | H | 0.12 |
| 163 | (Hopkins et al., 2019) | H | Cl | H | 1.8 |
| 164 | (Hopkins et al., 2019) | H | H | Cl | 2.3 |
| 165 | (Hopkins et al., 2019) | CF_3_ | H | H | 0.46 |

| **Table B:** Training subsets employed in exploring the pharmacophoric space of BTK inhibitors. | | | | |
| --- | --- | --- | --- | --- |
| **Set** | **Parent compound** | **Feature map** | **Active compounds ^a,c^** | **Intermediate and**  **Inactive compounds ^b^** |
| **A** | **34**  (IC_50_ = 2 nM) | **A** | 2, 4, 5, 6, 10, 21, 24, 25, 26, 31, **34**, 36, 37, 38, 39, 40, 41, 42, 43, 44, 45, 46, 79, 80, 81, 83, 84, 85, 86, 87, 88, 106, 135, 141, 142 | 1, 8, 9, 11, 12, 13, 14, 15, 16, 17, 18, 19, 20, 23, 30, 32, 33, 35, 78, 89, 90, 91, 92, 93, 94, 95, 96, 97, 98, 99, 100, 101, 102, 103, 104, 105, 107, 108, 109, 110, 111, 112, 113, 114, 115, 116, 117, 118, 119, 121, 122, 123, 124, 125, 138, 140, 143, 144, 145, 146, 147, 148, 149, 150, 152, 153, 154, 156, 157, 158, 159, 160, 161, 162, 163, 164, 165 |
| **B** | **31**  (IC_50_ = 5.1 nM) | **B** | 2, 6, 10, 24, 25, 26, **31**, 36, 37, 40, 41, 42, 44, 45, 46, 83, 85 | 1, 8, 11, 12, 13, 15, 16, 20, 30, 35, 78, 89, 91, 96, 98, 99, 103, 104, 105, 107, 108, 111, 114, 117, 118, 119, 125, 138, 145, 146, 147, 148, 149, 150, 153, 156, 157, 158, 160, 161, 163, 164 |
| **C** | **6**  (IC_50_ = 1.9 nM) | **C** | **6**, 7, 10, 21, 24, 25, 26, 31, 36, 44, 45, 46, 83, 84, 85, 87 | 1, 8, 15, 35, 91, 94, 95, 98, 99, 101, 102, 103, 104, 107, 108, 113, 115, 116, 117, 118, 119, 123, 124, 125, 145, 146, 147, 148, 150, 153, 156, 157, 158, 161, 164 |
| **D** | **80**  (IC_50_ = 1.6 nM) | **D** | 79, **80**, 82, 106, 132, 135, 136, 137, 141, 142 | 17, 94, 99, 100, 102, 112, 114, 115, 123, 124, 125, 140, 143, 144, 145, 146, 147, 150, 151, 153, 158 |
| **E** | **82**  (IC_50_ = 2.5 nM) | **E** | 37, 39, 40, 41, 43, 46, 58, 71, 81, **82** | 30, 57, 59, 60, 70, 78, 91, 92, 95, 99, 101, 103, 138, 155 |
| **F** | **106**  (IC_50_ = 8 nM) | **F** | 38, 43, 45, **106** | 90, 91, 92, 93, 94, 95, 96, 97, 99, 101, 103, 104, 107, 108, 111, 112, 113, 114, 115, 116, 117, 118, 122, 123, 124, 164 |
| **G** | **131**  (IC_50_ = 4 nM) | **G** | 21, 24, 25, 26, 31, 34, 38, 88, 106, **131**, 132, 134, 141, 142 | 11, 12, 15, 16, 30, 35, 89, 90, 91, 92, 93, 94, 95, 96, 97, 98, 99, 100, 102, 104, 105, 107, 108, 112, 113, 114, 115, 116, 117, 118, 119, 120, 122, 123, 124, 125, 127, 128, 129, 130, 133, 138, 140, 143, 144, 145, 146, 147, 148, 149, 150, 152, 153, 154, 156, 157, 158, 159, 160, 161, 162, 163, 164, 165 |
| **H** | **36**  (IC_50_ = 7.2 nM) | **H** | 2, 4, 5, 6, **36**, 38, 40, 42, 43, 44, 45, 84, 85, 86, 87, 88 | 32, 33, 35, 90, 91, 94, 95, 98, 99, 103, 104, 107, 108, 115, 116, 117, 118, 119, 124, 125, 146, 147, 148, 150, 156, 157, 158 |

**^a^** Catalyst identifies a specific compound as being within the most active category if it satisfies the Equation:

(Activity of most active x Uncertainty of most active) - (Activity of questioned compound / Uncertainty of questioned compound) > 0.0

**^b^** Catalyst identifies a specific compound as being within the Inactive category if it satisfies the Equation:

Log (Activity of questioned compound) – Log (Activity of most active compound) > 3.5

**^c^** Most active compounds are bolded.

**Table C**: Training sets and CATALYST run parameters employed for exploring BTK pharmacophoric space.

| **Run**  **Number** | **Training**  **set^a^** | **Number of**  **compounds** | **Selected features^b^** | **Max-Min**  **Features^c^** | **Spacing^d^** |
| --- | --- | --- | --- | --- | --- |
| 1  2  3  4  5 | **A** | 112 | Hbic (0-5), HBA (0-3), HBD (0-3), NegIon (0-3), PosCha (0-3) | 4-5 | 1 |
| 2 |  | 112 | Hbic (0-5), HBA (0-3), HBD (0-3), NegIon (0-3), PosCha (0-3) | 5-5 | 1 |
| 3 |  | 112 | Hbic (0-5), HBA (0-3), HBD (0-3), NegIon (0-3), PosCha (0-3) | 4-5 | 3 |
| 4 |  | 112 | Hbic (0-5), HBA (0-3), HBD (0-3), NegIon (0-3), PosCha (0-3) | 5-5 | 3 |
| 5 |  | 112 | Hbic (0-5), HBA (0-3), HBD (0-3), NegIon (0-3), PosCha (0-3), Exvl (0-10) | 4-5 | 1 |
| 6 |  | 112 | Hbic (0-5), HBA (0-3), HBD (0-3), NegIon (0-3), PosCha (0-3), Exvl (0-10) | 5-5 | 1 |
| 7 |  | 112 | Hbic (0-5), HBA (0-3), HBD (0-3), NegIon (0-3), PosCha (0-3), Exvl (0-10) | 4-5 | 3 |
| 8 |  | 112 | Hbic (0-5), HBA (0-3), HBD (0-3), NegIon (0-3), PosCha (0-3), Exvl (0-10) | 5-5 | 3 |
| 1  2  3  4  5 | **B** | 59 | Hbic (0-5), HBA (0-3), HBD (0-3), NegIon (0-3), PosCha (0-3) | 4-5 | 1 |
| 2 |  | 59 | Hbic (0-5), HBA (0-3), HBD (0-3), NegIon (0-3), PosCha (0-3) | 5-5 | 1 |
| 3 |  | 59 | Hbic (0-5), HBA (0-3), HBD (0-3), NegIon (0-3), PosCha (0-3) | 4-5 | 3 |
| 4 |  | 59 | Hbic (0-5), HBA (0-3), HBD (0-3), NegIon (0-3), PosCha (0-3) | 5-5 | 3 |
| 5 |  | 59 | Hbic (0-5), HBA (0-3), HBD (0-3), NegIon (0-3), PosCha (0-3), Exvl (0-10) | 4-5 | 1 |
| 6 |  | 59 | Hbic (0-5), HBA (0-3), HBD (0-3), NegIon (0-3), PosCha (0-3), Exvl (0-10) | 5-5 | 1 |
| 7 |  | 59 | Hbic (0-5), HBA (0-3), HBD (0-3), NegIon (0-3), PosCha (0-3), Exvl (0-10) | 4-5 | 3 |
| 8 |  | 59 | Hbic (0-5), HBA (0-3), HBD (0-3), NegIon (0-3), PosCha (0-3), Exvl (0-10) | 5-5 | 3 |
| 1  2  3  4  5 | **C** | 51 | Hbic (0-5), HBA (0-3), HBD (0-3), NegIon (0-3), PosCha (0-3) | 4-5 | 1 |
| 2 |  | 51 | Hbic (0-5), HBA (0-3), HBD (0-3), NegIon (0-3), PosCha (0-3) | 5-5 | 1 |
| 3 |  | 51 | Hbic (0-5), HBA (0-3), HBD (0-3), NegIon (0-3), PosCha (0-3) | 4-5 | 3 |
| 4 |  | 51 | Hbic (0-5), HBA (0-3), HBD (0-3), NegIon (0-3), PosCha (0-3) | 5-5 | 3 |
| 5 |  | 51 | Hbic (0-5), HBA (0-3), HBD (0-3), NegIon (0-3), PosCha (0-3), Exvl (0-10) | 4-5 | 1 |
| 6 |  | 51 | Hbic (0-5), HBA (0-3), HBD (0-3), NegIon (0-3), PosCha (0-3), Exvl (0-10) | 5-5 | 1 |
| 7 |  | 51 | Hbic (0-5), HBA (0-3), HBD (0-3), NegIon (0-3), PosCha (0-3), Exvl (0-10) | 4-5 | 3 |
| 8 |  | 51 | Hbic (0-5), HBA (0-3), HBD (0-3), NegIon (0-3), PosCha (0-3), Exvl (0-10) | 5-5 | 3 |
| 1  2  3  4  5 | **D** | 31 | Hbic (0-5), HBA (0-3), HBD (0-3), NegIon (0-3), PosCha (0-3) | 4-5 | 1 |
| 2 |  | 31 | Hbic (0-5), HBA (0-3), HBD (0-3), NegIon (0-3), PosCha (0-3) | 5-5 | 1 |
| 3 |  | 31 | Hbic (0-5), HBA (0-3), HBD (0-3), NegIon (0-3), PosCha (0-3) | 4-5 | 3 |
| 4 |  | 31 | Hbic (0-5), HBA (0-3), HBD (0-3), NegIon (0-3), PosCha (0-3) | 5-5 | 3 |
| 5 |  | 31 | Hbic (0-5), HBA (0-3), HBD (0-3), NegIon (0-3), PosCha (0-3), Exvl (0-10) | 4-5 | 1 |
| 6 |  | 31 | Hbic (0-5), HBA (0-3), HBD (0-3), NegIon (0-3), PosCha (0-3), Exvl (0-10) | 5-5 | 1 |
| 7 |  | 31 | Hbic (0-5), HBA (0-3), HBD (0-3), NegIon (0-3), PosCha (0-3), Exvl (0-10) | 4-5 | 3 |
| 8 |  | 31 | Hbic (0-5), HBA (0-3), HBD (0-3), NegIon (0-3), PosCha (0-3), Exvl (0-10) | 5-5 | 3 |
| 1  2  3  4  5 | **E** | 24 | Hbic (0-5), HBA (0-3), HBD (0-3), NegIon (0-3), PosCha (0-3) | 4-5 | 1 |
| 2 |  | 24 | Hbic (0-5), HBA (0-3), HBD (0-3), NegIon (0-3), PosCha (0-3) | 5-5 | 1 |
| 3 |  | 24 | Hbic (0-5), HBA (0-3), HBD (0-3), NegIon (0-3), PosCha (0-3) | 4-5 | 3 |
| 4 |  | 24 | Hbic (0-5), HBA (0-3), HBD (0-3), NegIon (0-3), PosCha (0-3) | 5-5 | 3 |
| 5 |  | 24 | Hbic (0-5), HBA (0-3), HBD (0-3), NegIon (0-3), PosCha (0-3), Exvl (0-10) | 4-5 | 1 |
| 6 |  | 24 | Hbic (0-5), HBA (0-3), HBD (0-3), NegIon (0-3), PosCha (0-3), Exvl (0-10) | 5-5 | 1 |
| 7 |  | 24 | Hbic (0-5), HBA (0-3), HBD (0-3), NegIon (0-3), PosCha (0-3), Exvl (0-10) | 4-5 | 3 |
| 8 |  | 24 | Hbic (0-5), HBA (0-3), HBD (0-3), NegIon (0-3), PosCha (0-3), Exvl (0-10) | 5-5 | 3 |

^a^Training subsets as in Table 2.

^b^HBA: Hydrogen Bond Acceptor, HBD: Hydrogen Bond Donor, Hbic: Hydrophobic, NegIon: Negative Ionizable, Exvl: Exclusion volumes, PosCha: Positive Charge. Numbers in brackets refer to the allowed ranges of corresponding features in each pharmacophore modeling run.

^c^Max-Min refers to the allowed range of pharmacophoric features in each model.

**Table C**: Training sets and CATALYST run parameters employed for exploring BTK pharmacophoric space.

| **Run**  **Number** | **Training**  **set^a^** | **Number of**  **compounds** | **Selected features^b^** | **Max-Min**  **Features^c^** | **Spacing^d^** |
| --- | --- | --- | --- | --- | --- |
| 1  2  3  4  5 | **F** | 30 | Hbic (0-5), HBA (0-3), HBD (0-3), NegIon (0-3), PosCha (0-3) | 4-5 | 1 |
| 2 |  | 30 | Hbic (0-5), HBA (0-3), HBD (0-3), NegIon (0-3), PosCha (0-3) | 5-5 | 1 |
| 3 |  | 30 | Hbic (0-5), HBA (0-3), HBD (0-3), NegIon (0-3), PosCha (0-3) | 4-5 | 3 |
| 4 |  | 30 | Hbic (0-5), HBA (0-3), HBD (0-3), NegIon (0-3), PosCha (0-3) | 5-5 | 3 |
| 5 |  | 30 | Hbic (0-5), HBA (0-3), HBD (0-3), NegIon (0-3), PosCha (0-3), Exvl (0-10) | 4-5 | 1 |
| 6 |  | 30 | Hbic (0-5), HBA (0-3), HBD (0-3), NegIon (0-3), PosCha (0-3), Exvl (0-10) | 5-5 | 1 |
| 7 |  | 30 | Hbic (0-5), HBA (0-3), HBD (0-3), NegIon (0-3), PosCha (0-3), Exvl (0-10) | 4-5 | 3 |
| 8 |  | 30 | Hbic (0-5), HBA (0-3), HBD (0-3), NegIon (0-3), PosCha (0-3), Exvl (0-10) | 5-5 | 3 |
| 1  2  3  4  5 | **G** | 78 | Hbic (0-5), HBA (0-3), HBD (0-3), NegIon (0-3), PosCha (0-3) | 4-5 | 1 |
| 2 |  | 78 | Hbic (0-5), HBA (0-3), HBD (0-3), NegIon (0-3), PosCha (0-3) | 5-5 | 1 |
| 3 |  | 78 | Hbic (0-5), HBA (0-3), HBD (0-3), NegIon (0-3), PosCha (0-3) | 4-5 | 3 |
| 4 |  | 78 | Hbic (0-5), HBA (0-3), HBD (0-3), NegIon (0-3), PosCha (0-3) | 5-5 | 3 |
| 5 |  | 78 | Hbic (0-5), HBA (0-3), HBD (0-3), NegIon (0-3), PosCha (0-3), Exvl (0-10) | 4-5 | 1 |
| 6 |  | 78 | Hbic (0-5), HBA (0-3), HBD (0-3), NegIon (0-3), PosCha (0-3), Exvl (0-10) | 5-5 | 1 |
| 7 |  | 78 | Hbic (0-5), HBA (0-3), HBD (0-3), NegIon (0-3), PosCha (0-3), Exvl (0-10) | 4-5 | 3 |
| 8 |  | 78 | Hbic (0-5), HBA (0-3), HBD (0-3), NegIon (0-3), PosCha (0-3), Exvl (0-10) | 5-5 | 3 |
| 1  2  3  4  5 | **H** | 43 | Hbic (0-5), HBA (0-3), HBD (0-3), NegIon (0-3), PosCha (0-3) | 4-5 | 1 |
| 2 |  | 43 | Hbic (0-5), HBA (0-3), HBD (0-3), NegIon (0-3), PosCha (0-3) | 5-5 | 1 |
| 3 |  | 43 | Hbic (0-5), HBA (0-3), HBD (0-3), NegIon (0-3), PosCha (0-3) | 4-5 | 3 |
| 4 |  | 43 | Hbic (0-5), HBA (0-3), HBD (0-3), NegIon (0-3), PosCha (0-3) | 5-5 | 3 |
| 5 |  | 43 | Hbic (0-5), HBA (0-3), HBD (0-3), NegIon (0-3), PosCha (0-3), Exvl (0-10) | 4-5 | 1 |
| 6 |  | 43 | Hbic (0-5), HBA (0-3), HBD (0-3), NegIon (0-3), PosCha (0-3), Exvl (0-10) | 5-5 | 1 |
| 7 |  | 43 | Hbic (0-5), HBA (0-3), HBD (0-3), NegIon (0-3), PosCha (0-3), Exvl (0-10) | 4-5 | 3 |
| 8 |  | 43 | Hbic (0-5), HBA (0-3), HBD (0-3), NegIon (0-3), PosCha (0-3), Exvl (0-10) | 5-5 | 3 |

^a^Training subsets as in Table 2.

^b^HBA: Hydrogen Bond Acceptor, HBD: Hydrogen Bond Donor, Hbic: Hydrophobic, NegIon: Negative Ionizable, Exvl: Exclusion volumes, PosCha: Positive Charge. Numbers in brackets refer to the allowed ranges of corresponding features in each pharmacophore modeling run.

^c^Max-Min refers to the allowed range of pharmacophoric features in each model.

|  |  | |  |
| --- | --- | --- | --- |
| Compound 34, IC_50_ = 2 nM, Set A | Compound 31, IC_50_ = 5.1 nM, Set B | | Compound 6, IC_50_ = 1.9 nM, Set C |
|  |  | |  |
| Compound 80, IC_50_ = 1.6 nM, Set D | Compound 82, IC_50_ = 2.5 nM, Set E | | Compound 106, IC_50_ = 8 nM, Set F |
|  | |  | |
| Compound 131, IC_50_ = 4 nM, Set G | | Compound 36, IC_50_ = 7.2 nM, Set H | |

**Figure A: Template compounds and the pharmacophoric maps used to build the subsets.** 3D structures of selected diverse and potent compounds that served as templates. Each structure is shown with superimposed pharmacophoric features represented by colored spheres: green (hydrophobic), red (positive ionizable), cyan (hydrogen bond donor), and purple (aromatic ring). Below each structure, the compound identifier according to table A, and their experimentally determined activity value in nanomolar (nM). The compounds are organized into distinct Sets labeled A, B, C, D, E, F, G, and H. These template compounds were identified from the dataset; their source and further details can be found in Table A in the Supporting Material. These compounds and their pharmacophoric representations were utilized as templates for identifying similar molecules in the dataset, following the "Diversity-Driven Subset Generation" technique that is detailed in Supporting Information Section S2.

| Compound **166**, Cell line K562, IC_50_ = 0.73 µM | Compound **166**, Cell line Raji, IC_50_ = 0.087 µM |
| --- | --- |
|  |  |
| Compound **167**, Cell line K562, IC_50_ = 8.638 µM | Compound **167**, Cell line Raji, IC_50_ = 5.756 µM |
|  |  |
| Compound **168**, Cell line K562, IC_50_ = 4.37 µM | Compound **168**, Cell line Raji, IC_50_ = 4.92 µM |

**Figure B: Dose-Inhibition plots against of BTK active inhibitors against different cell lines.**

| Compound **169**, Cell line K562, IC_50_ = 6.18 µM | Compound **169**, Cell line Raji, IC_50_ = 12.73 µM |
| --- | --- |
|  |  |
| Compound **170**, Cell line K562, IC_50_ = 11.13 µM | Compound **170**, Cell line Raji, IC_50_ = 21.78 µM |
|  |  |
| Compound **171**, Cell line K562, IC_50_ = 4.26 µM | Compound **171**, Cell line Raji, IC_50_ = 11.23 µM |

**Figure B: Dose-Inhibition plots against of BTK active inhibitors against different cell lines.**

| Compound **172**, Cell line K562, IC_50_ = 9.29 µM | Compound **172**, Cell line Raji, IC_50_ = 27.34 µM |
| --- | --- |
|  |  |
| Compound **173**, Cell line K562, IC_50_ = 47.68 µM | Compound **173**, Cell line Raji, IC_50_ = ND |
|  |  |
| Compound **174**, Cell line K562, IC_50_ = ND | Compound **174**, Cell line Raji, IC_50_ = 53.27 µM |

**Figure B: Dose-Inhibition plots against of BTK active inhibitors against different cell lines.**

**Figure C**: ^1^HNMR spectrum of NCI hit compound **TK2** (Table 6, NCI code: **604558**).

**Figure D:** Correlation between predicted and experimentally determined IC₅₀ values across two prediction frameworks and two cellular models. Scatter plots show log₁₀-transformed predicted IC₅₀ values from the ML-RF(PCA) and GFA-MLR models plotted against experimentally measured IC₅₀ values in Raji (CLL lymphoma) cells (A,B) and K562 (leukemia) cells (C,D). Panels correspond to (A) Raji–ML-RF(PCA), (B) Raji–GFA-MLR, (C) K562–ML-RF(PCA), and (D) K562–GFA-MLR. Spearman rank correlation coefficients (ρ) are indicated in each panel.

**Figure S1.** Superposition of the crystallographic ligand (green) and the re-docked pose (orange) in the BTK ATP-binding site (PDB ID 5T18). The docking protocol reproduced the experimental binding mode with an RMSD of 0.83 Å.

**References**

Caldwell, R., Liu-Bujalski, L., Qiu, H., Mochalkin, I., Jones, R., Neagu, C., Goutopoulos, A., Grenningloh, R., Johnson, T., Sherer, B., Gardberg, A., Follis, A. V., Morandi, F., & Head, J. (2018). Discovery of a novel series of pyridine and pyrimidine carboxamides as potent and selective covalent inhibitors of Btk. *Bioorganic & Medicinal Chemistry Letters*, *28*(21), 3419–3424. https://doi.org/10.1016/j.bmcl.2018.09.033

Crawford, J. J., Lee, W., Johnson, A. R., Delatorre, K. J., Chen, J., Eigenbrot, C., Heidmann, J., Kakiuchi-Kiyota, S., Katewa, A., Kiefer, J. R., Liu, L., Lubach, J. W., Misner, D., Purkey, H., Reif, K., Vogt, J., Wong, H., Yu, C., & Young, W. B. (2020). Stereochemical Differences in Fluorocyclopropyl Amides Enable Tuning of Btk Inhibition and Off-Target Activity. *ACS Medicinal Chemistry Letters*, *11*(8), 1588–1597. https://doi.org/10.1021/acsmedchemlett.0c00249

de Bruin, G., Demont, D., de Zwart, E., Verkaik, S., Hoogenboom, N., van de Kar, B., van Lith, B., Emmelot- van Hoek, M., Gulrajani, M., Covey, T., Kaptein, A., & Barf, T. (2020). Discovery of quinoline-based irreversible BTK inhibitors. *Bioorganic & Medicinal Chemistry Letters*, *30*(14), 127261. https://doi.org/10.1016/j.bmcl.2020.127261

Hopkins, B. T., Bame, E., Bell, N., Bohnert, T., Bowden-Verhoek, J. K., Bui, M., Cancilla, M. T., Conlon, P., Cullen, P., Erlanson, D. A., Fan, J., Fuchs-Knotts, T., Hansen, S., Heumann, S., Jenkins, T. J., Marcotte, D., McDowell, B., Mertsching, E., Negrou, E., … Zhong, M. (2019). Optimization of novel reversible Bruton’s tyrosine kinase inhibitors identified using Tethering-fragment-based screens. *Bioorganic & Medicinal Chemistry*, *27*(13), 2905–2913. https://doi.org/10.1016/j.bmc.2019.05.021

Kim, K.-H., Maderna, A., Schnute, M. E., Hegen, M., Mohan, S., Miyashiro, J., Lin, L., Li, E., Keegan, S., Lussier, J., Wrocklage, C., Nickerson-Nutter, C. L., Wittwer, A. J., Soutter, H., Caspers, N., Han, S., Kurumbail, R., Dunussi-Joannopoulos, K., Douhan, J., & Wissner, A. (2011). Imidazo[1,5-a]quinoxalines as irreversible BTK inhibitors for the treatment of rheumatoid arthritis. *Bioorganic & Medicinal Chemistry Letters*, *21*(21), 6258–6263. https://doi.org/10.1016/j.bmcl.2011.09.008

Pan, Z., Scheerens, H., Li, S., Schultz, B. E., Sprengeler, P. A., Burrill, L. C., Mendonca, R. V., Sweeney, M. D., Scott, K. C. K., Grothaus, P. G., Jeffery, D. A., Spoerke, J. M., Honigberg, L. A., Young, P. R., Dalrymple, S. A., & Palmer, J. T. (2007). Discovery of Selective Irreversible Inhibitors for Bruton’s Tyrosine Kinase. *ChemMedChem*, *2*(1), 58–61. https://doi.org/10.1002/cmdc.200600221

Qiu, H., Liu-Bujalski, L., Caldwell, R. D., Viacava Follis, A., Gardberg, A., Goutopoulos, A., Grenningloh, R., Head, J., Johnson, T., Jones, C. C. V., Jones, R., Mochalkin, I., Morandi, F., Neagu, C., Potnick, J., & Sherer, B. (2018). Optimization of the efflux ratio and permeability of covalent irreversible BTK inhibitors. *Bioorganic & Medicinal Chemistry Letters*, *28*(20), 3307–3311. https://doi.org/10.1016/j.bmcl.2018.09.018

Shi, Q., Tebben, A., Dyckman, A. J., Li, H., Liu, C., Lin, J., Spergel, S., Burke, J. R., McIntyre, K. W., Olini, G. C., Strnad, J., Surti, N., Muckelbauer, J. K., Chang, C., An, Y., Cheng, L., Ruan, Q., Leftheris, K., Carter, P. H., … De Lucca, G. V. (2014). Purine derivatives as potent Bruton’s tyrosine kinase (BTK) inhibitors for autoimmune diseases. *Bioorganic & Medicinal Chemistry Letters*, *24*(9), 2206–2211. https://doi.org/10.1016/j.bmcl.2014.02.075

Smith, C. R., Dougan, D. R., Komandla, M., Kanouni, T., Knight, B., Lawson, J. D., Sabat, M., Taylor, E. R., Vu, P., & Wyrick, C. (2015). Fragment-Based Discovery of a Small Molecule Inhibitor of Bruton’s Tyrosine Kinase. *Journal of Medicinal Chemistry*, *58*(14), 5437–5444. https://doi.org/10.1021/acs.jmedchem.5b00734

Watterson, S. H., De Lucca, G. V., Shi, Q., Langevine, C. M., Liu, Q., Batt, D. G., Beaudoin Bertrand, M., Gong, H., Dai, J., Yip, S., Li, P., Sun, D., Wu, D.-R., Wang, C., Zhang, Y., Traeger, S. C., Pattoli, M. A., Skala, S., Cheng, L., … Tino, J. A. (2016). Discovery of 6-Fluoro-5-( *R* )-(3-( *S* )-(8-fluoro-1-methyl-2,4-dioxo-1,2-dihydroquinazolin-3(4 *H* )-yl)-2-methylphenyl)-2-( *S* )-(2-hydroxypropan-2-yl)-2,3,4,9-tetrahydro-1 *H* -carbazole-8-carboxamide (BMS-986142): A Reversible Inhibitor of Bruton’s Tyrosine Kinase (BTK) Conformationally Constrained by Two Locked Atropisomers. *Journal of Medicinal Chemistry*, *59*(19), 9173–9200. https://doi.org/10.1021/acs.jmedchem.6b01088
